# Pd/Ligand-Free Synthesis of 2-Alkynylated Pyrano[4,3-*d*]imidazol-4-ones via One-Pot Cu-Mediated Tandem Sonogashira Coupling/Regioselective 6-*endo*-*dig* Oxacyclization Reaction

**DOI:** 10.3390/molecules30143045

**Published:** 2025-07-21

**Authors:** Abir Ayachi, Abdellatif Tikad, Vincent Lazeran, Hassan Allouchi, Marc Bletry, Rafâa Besbes, Mohamed Abarbri, Badr Jismy

**Affiliations:** 1 Laboratoire de Physico-Chimie des Matériaux et des Electrolytes Pour l’Energie (PCM2E), EA 6299, Faculté des Sciences et Techniques, Avenue Monge, Faculté des Sciences, Université de Tours, Parc de Grandmont, 37200 Tours, France; abir.ayachi@etu.univ-tours.fr (A.A.); lazeran@univ-tours.fr (V.L.); marc.bletry@univ-tours.fr (M.B.); mohamed.abarbri@univ-tours.fr (M.A.); 2 Laboratoire de Chimie Analytique et Electrochimie (LCAE), Faculté des Sciences de Tunis, Université de Tunis El Manar, Campus Universitaire, El-Manar, Tunis 2092, Tunisia; rafaa.besbes@fst.utm.tn; 3 Laboratoire de Chimie Moléculaire et Substances Naturelles, Faculté des Sciences, Université Moulay Ismail, B.P. 11201, Zitoune, Meknès 50050, Morocco; a.tikad@umi.ac.ma; 4 Laboratoire de Synthèse et Isolement de Molécules BioActives, SIMBA EA 7502, Faculté de Pharmacie, Université de Tours, 31 Avenue Monge, 37200 Tours, France; hassan.allouchi@univ-tours.fr

**Keywords:** Sonogashira cross-coupling, copper catalysis, 6-*endo*-*dig* oxacyclization, pyrano[4,3-*d*]imidazol-4-one, computational study, DFT

## Abstract

Herein, we report a one-pot palladium- and ligand-free tandem Sonogashira coupling/regioselective 6-*endo*-*dig* oxacyclization reaction of 2,4-diiodo-1-methyl-imidazole-5-carboxylic acid with terminal alkynes mediated by Copper(I). This impressive approach offers a straightforward, practical, and efficient tandem procedure for accessing 2-alkynylated pyrano[4,3-*d*]imidazol-4-one in moderate to good yields with an exclusive 6-*endo*-*dig* oxacyclization. Notably, this cost-effective methodology demonstrates broad substrate compatibility with various commercially available aliphatic and (hetero)aromatic terminal alkynes. Furthermore, DFT studies were performed to elucidate the origin of this regioselective 6-*endo*-*dig* oxacyclization reaction.

## 1. Introduction

**Figure 1:**
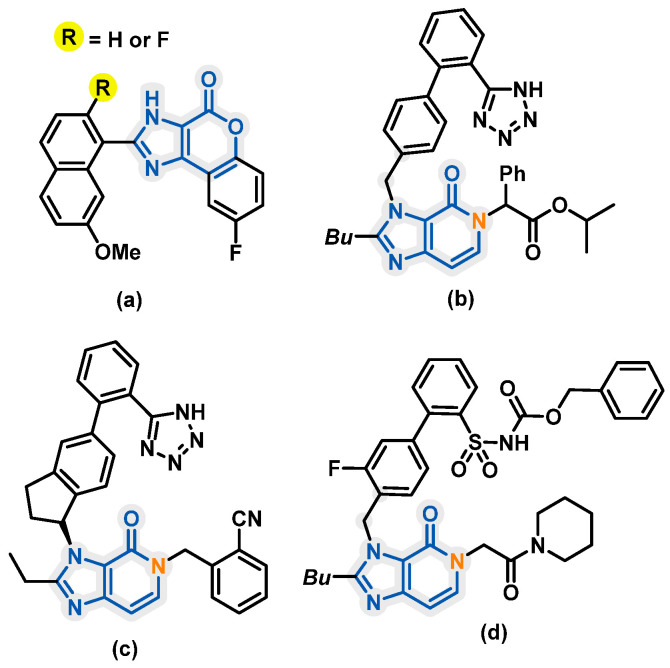
Some examples of bioactive compounds containing the pyrano[4,3-*d*]imidazol-4-one skeleton and derivatives. (**a**) DNA gyrase and topo IV inhibitor; (**b**–**d**) antagonists of the angiotensin II.

2*H*-Pyran-2-ones, otherwise known as 2-pyrones or 2-pyranones (α-pyrones), are six-membered heterocyclic esters embedding an oxygen atom and a carbonyl group. They are frequently encountered in a large number of natural products, pharmaceutical compounds, and other functional molecules, displaying a broad spectrum of important bioactive properties [1,2]. Particularly, fused 2*H*-pyran-2-ones with a five-membered heteroaryl ring, which are undoubtedly of great value because of their own structural diversity and biological properties, have garnered widespread attention from the chemical research community [3,4,5]. Pyrano[4,3-*d*]imidazol-4-one derivatives are typical examples of these types of compounds, which have also demonstrated notable bioactivities, including the inhibition of bacterial DNA gyrase and topoisomerase IV (Figure 1a) [6], antagonists of the angiotensin II receptor with different affinities for the AT1 and AT2 subtypes (Figure 1b–d) [7,8,9], as well as partial agonists of peroxisome proliferator-activated receptor-γ (PPARγ) (Figure 1c) [9].

**Figure 2:**
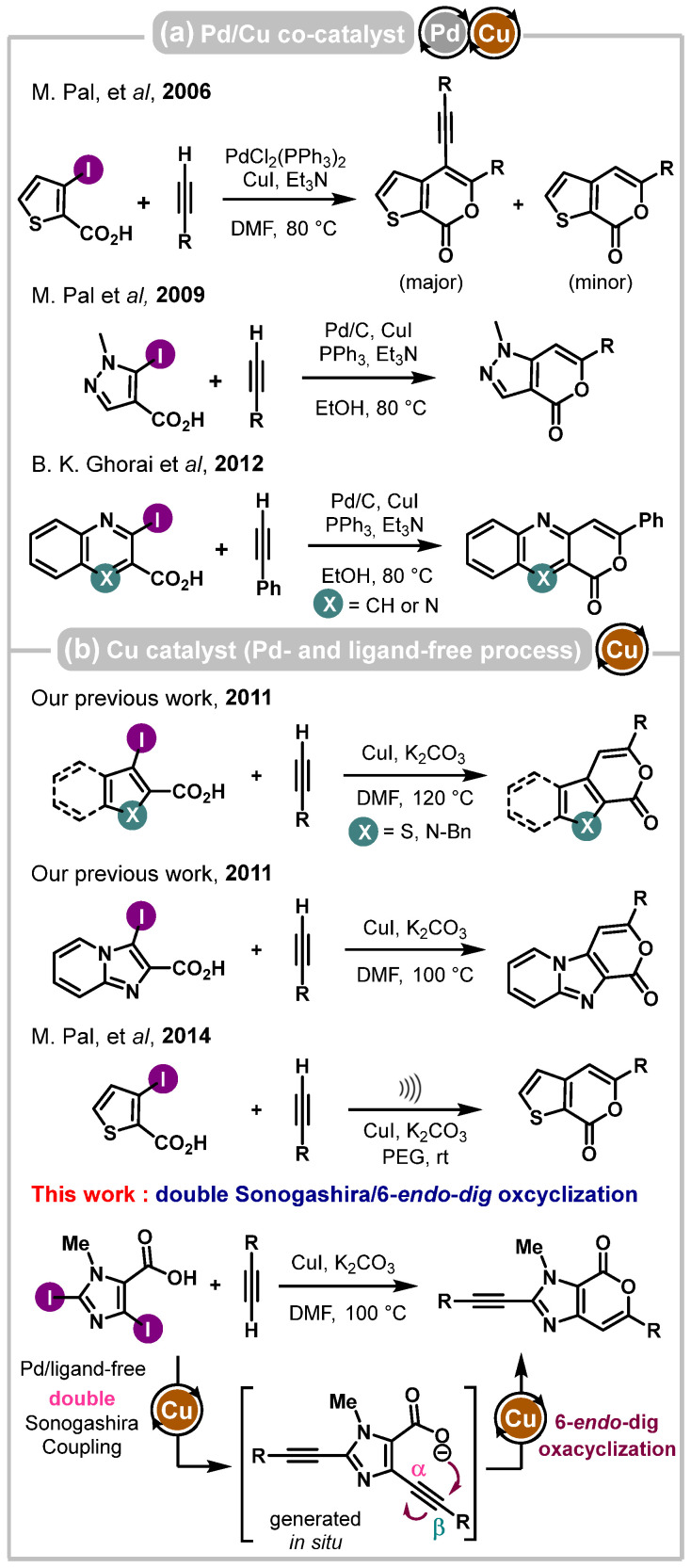
Previous and present work on Sonogashira/6-*endo*-*dig* cyclization of different heteroaromatic β-iodo-α,β-unsaturated carboxylic acid. (**a**) Pd/Cu co-catalyst process developed by M. Pal et al. (2006) [10], M. Pal et al. (2009) [11] and by B. K. Ghorai et al. (2012) [12]; (**b**) Cu catalyst process developed by our group (2011) [14,15] and by M. Pal et al. (2014) [13].

Given the diverse biological properties of 2*H*-pyran-2-ones derivatives, the development of practical and highly efficient synthetic methods is still in high demand. Although significant efforts have been devoted to the synthesis of these heterocycles, only a few synthetic methods for the preparation of 2*H*-pyran-2-ones fused heterocyclic systems have been reported. Among them, the one-pot tandem Sonogashira coupling/oxacyclization reaction from heteroaromatic β-iodo/bromo-α,β-unsaturated carboxylic acid derivatives and terminal alkynes has emerged as a general approach to obtain these valuable compounds. However, this transformation generally requires the use of expensive palladium catalysts accompanied by copper as a cocatalyst, including ligand effects in some cases, which significantly limits the usefulness of these conditions (Figure 2a) [10,11,12]. In the last decade, our group has developed a new versatile, direct, and elegant method under palladium- and ligand-free conditions, using exclusively copper(I) as a cheaper catalyst. Moreover, this protocol provides novel, facile, rapid, and efficient access to structurally diverse bi- and tri-heterocyclic 2*H*-pyran-2-ones based on thiophene [13,14], indole [14], and imidazo[1,2-*a*]pyridine [15] rings in a highly atom-economical manner (Figure 2b).

It is widespread recognition that the Sonogashira cross-coupling reaction is routinely conducted with a palladium-based catalyst and a copper(I) salt as a cocatalyst [16]. Although numerous different copper-free, palladium-based catalytic systems have been recently established for this reaction [17,18], efficient palladium- and ligand-free copper-based catalytic systems have remained relatively underexplored (Figure 2b), even though they are obviously be much more interesting in terms of environmental and economic points of view than palladium-based ones [19].

In light of the diverse biological properties of 2*H*-pyran-2-ones derivatives and fascinated by the great features of Sonogashira cross-coupling catalyzed exclusively by copper, which are cheaper, more abundant, and more environmentally benign than those catalyzed by Pd complexes, herein we report an unprecedented one-pot copper-mediated tandem Sonogashira coupling/regioselective 6-*endo*-*dig* oxacyclization reaction of 2,4-diiodo-1-methyl-1*H*-imidazole-5-carboxylic acid **4** with terminal alkynes (Figure 2b).

Contrary to previous proposals, our approach offers notable improvements in terms of environmental impact, atom economy, efficiency, and product selectivity when compared to traditional and alternative literature-reported methods. Additionally, this strategy avoided the use of precious metal and ligand, with a drastic reduction in catalyst toxicity.

Our methodology offers a potential simplified approach to the synthesis of alkynylated isocoumarins, which are valuable building blocks for the preparation of an important class of antibiotics such as Rubromycin-type compounds [20,21,22] while circumventing multiple synthetic steps and eliminating the need for palladium catalysts. In addition to the synthetic utility of our methodology, the imidazole fused 2*H*-pyran-2-one structural motif present in the synthesized compounds is of considerable interest in medicinal chemistry, due to the significant biological activities exhibited by 2*H*-pyran-2-ones fused with five-membered heteroaryl rings, including antileishmanial, antifungal, and anticancer properties [3,4,5]. Future work will focus on the biological evaluation of these compounds to fully explore their therapeutic potential, as well as on expanding the scope of this methodology for the synthesis of natural products, pharmaceutically relevant compounds, and a broader range of heterocyclic scaffolds.

## 2. Results and Discussion

**Scheme 1:**
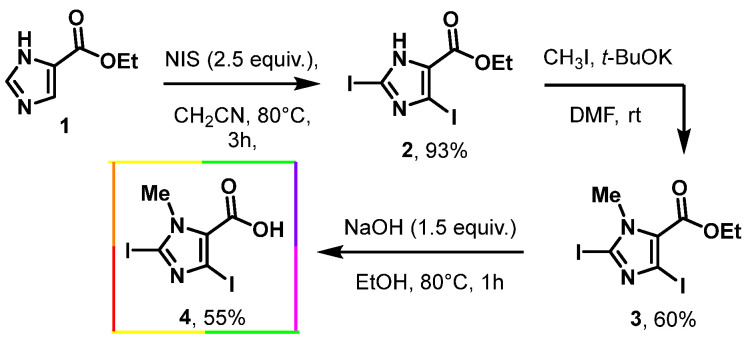
Synthesis of 2,4-diiodo-1-methyl-1*H*-imidazole-5-carboxylic acid **4** from ethyl 1*H*-imidazole-5-carboxylate **1**.

At the outset of our studies, 2,4-diiodo-1-methyl-1*H*-imidazole-5-carboxylic acid **4**, a five-membered *N*-heterocycle bearing a β-iodo-α,β-unsaturated carboxylic acid, was readily prepared in three steps on a multigram scale, as depicted in Scheme 1.

First, the intermediate **2** was synthesized in 93% yield via the bisiodination reaction of commercially available ethyl imidazole-4-carboxylate **1** using *N*-iodosuccinimide (NIS). The subsequent reaction of **2** with methyl iodide leads to regioselective methylation of the N1 position, yielding the desired product **3** in 60% yield. The synthesis was completed by the saponification reaction of ethyl ester **3** in the presence of sodium hydroxide, providing the corresponding carboxylic acid, the key intermediate **4**, in 55% yield.

Since the main goal of this study relies on the one-pot Cu-mediated tandem Sonogashira coupling/regioselective 6-*endo*-*dig* oxacyclization reaction, 2,4-diiodo-1-methyl-1*H*-imidazole-5-carboxylic acid **4** and phenylacetylene were selected as the model substrates to optimize the reaction conditions. In order to find the optimal conditions, the coupling partners were subjected to an extensive screening of several reaction parameters, such as catalyst and base, in DMF as a commonly used solvent for this reaction. Detailed information is summarized in Table 1.

We began our investigations with reaction conditions based on the work of Pal [10] and their co-workers using a Pd/Cu co-catalytic system (Table 1, entry 1). The reaction did not go to full conversion since only a mixture of desired product **5a** and the starting material **4** was observed in this case in 47:53 ratio. Furthermore, increasing the amount of copper iodide to 1.0 equivalent improved the desired product ratio **5a** (**5a**/**4** 70:30) but did not achieve the complete conversion of starting material **4** (Table 1, entry 2). Interestingly, the use of a mild inorganic base (K_2_CO_3_) instead of an organic base (Et_3_N) resulted in a full conversion of **4** after 3 h, providing the target product **5a** in 44% isolated yield (Table 1, entry 3). Encouraged by this promising result, we then examined whether both palladium and copper were essential to the success and efficiency of this reaction. Therefore, in the absence of palladium, the reaction afforded the desired product **5a** in just 27% yield after 24h, indicating that the reaction can proceed without palladium despite the low yield (Table 1, entry 4).

Gratifyingly, when the amount of CuI was increased to 2.0 equiv., the isolated yield of **5a** was improved to 51% after 3 h (Table 1, entry 5). Thereafter, the isolated yield of **5a** was slightly decreased to 47% when 2.5 equiv. of CuI was used as the catalyst loading (Table 1, entry 6). Unsurprisingly, when the alkyne loading was reduced to 2.05 and 1.05, no change was noted in the selectivity of the reaction, but the isolated yields decreased drastically to 24% and 12%, respectively (Table 1, entries 7–8). Subsequently, the influence of temperature on the reaction outcome was evaluated, but no further improvement in reaction yield was observed (Table 1, entries 9–10). Finally, switching from K_2_CO_3_ to various bases such as Na_2_CO_3_, K_3_PO_4_, Cs_2_CO_3,_ or Et_3_N did not give better reaction results (Table 1, entries 11–14).

**Scheme 2:**
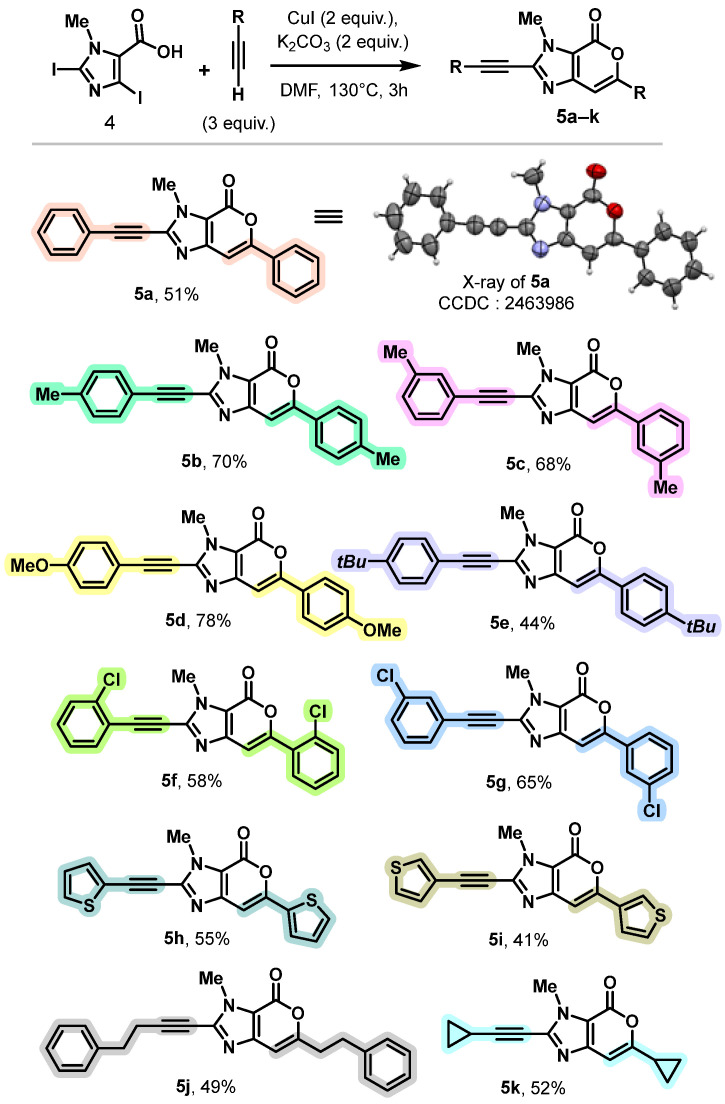
Substrate scope for the synthesis of pyrano[4,3-*d*]imidazol-4-one derivatives **5a**–**k**.

After successfully establishing the optimal reaction conditions (Table 1, entry 5), we evaluated the reaction scope and functional group tolerance against the catalytic system. Various commercially available aliphatic and (hetero)aromatic terminal alkynes reacted with 2,4-diiodo-1-methyl-1*H*-imidazole-5-carboxylic acid **4**, giving the desired products **5a**–**k** in 41–78% yields (Scheme 2).

The structure of **5a** was determined through comprehensive NMR and HRMS spectroscopic analyses and unambiguously confirmed by the X-ray crystallography of the single crystal (CCDC 2463986) (see Scheme 2) [23].

Reactions of **4** with phenyl-substituted acetylenes containing a methyl (Me) group at the para and meta positions proceeded smoothly under the well-established reaction conditions, providing the corresponding desired products **5b** and **5c** in 70% and 68% yields, respectively. It is noteworthy that the reaction of the phenyl substituent bearing an electron-donating group such as methoxy and *tert*-butyl at the para position worked efficiently in the current protocol, generating the expected products **5d** and **5e** in 78% and 44% yields, respectively. To our delight, the chloro-containing group at ortho and meta positions was a compatible functional group for this transformation, giving the desired products **5f** and **5g** in 58% and 65% yields, respectively. More importantly, heteroaromatic substrates such as 2-ethynylthiophene and 3-ethynylthiophene were also suitable substrates for this methodology and afforded the desired pyrano[4,3-*d*]imidazol-4-ones **5h** and **5i** in 55% and 41% yields, respectively. Interestingly, phenethylacetylene and cyclopropylacetylene, two aliphatic alkynes, were successfully reacted with **4**, leading the corresponding targeted products **5j** and **5k** in 49% and 52% yields, respectively.

**Scheme 3:**
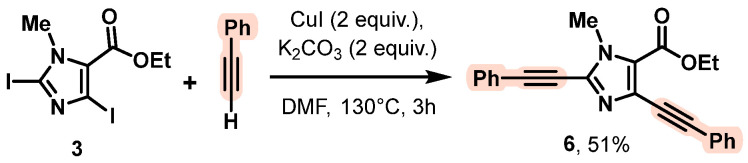
Copper-mediated Sonogashira reaction at C-2 and C-4 positions between 3 and phenylacetylene.

In the course of our investigation on the mechanistic evidence of this transformation, an additional experiment was performed. Under the same standard conditions, the reaction carried out with the intermediate **3** bearing an ester group instead of 2,4-diiodo-1-methyl-1*H*-imidazole-5-carboxylic acid **4**, exclusively affording 2,6-dialkynylated imidazole 6 instead of **5a** in 51% yield, as shown in Scheme 3.

This result indicates that, unlike the carboxylic acid function, the ester group of **3** was unable to participate in the regioselective 6-*endo*-*dig* oxacyclization step under the optimized conditions (Table 1, entry 5).

**Scheme 4:**
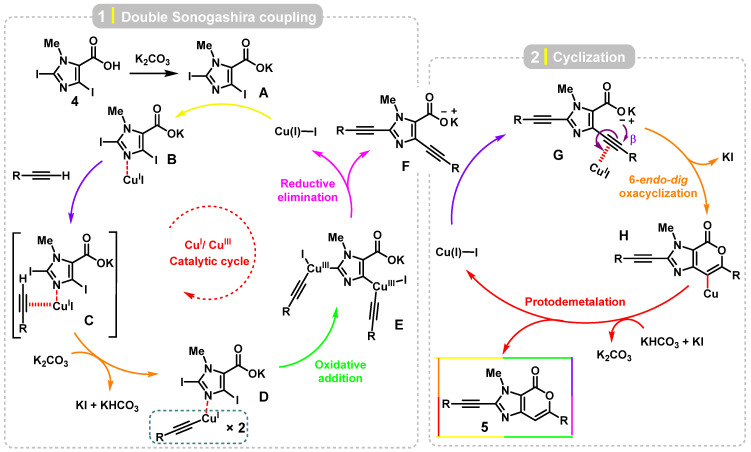
Plausible mechanism of the Cu-mediated tandem Sonogashira coupling/regioselective 6-*endo*-*dig* oxacyclization.

Based on similar studies reported in the literature [24,25,26] and the experiment performed before, a plausible mechanism of this transformation is proposed as shown in Scheme 4.

In the presence of the base K_2_CO_3_, the carboxylic acid **4** leads to the formation of potassium carboxylate **A**. Then, the nitrogen (*N*^X^) atom of imidazole, the nitrogen-based intra-ligand, coordinates the copper to form intermediate **B**. This species coordinates alkyne, and the deprotonated form of alkyne reacts with the Cu(I)–*N*(imidazole) ligand complex to provide the Cu(I)–acetylide species **D**. Next, the oxidative addition of heteroaryl iodide to intermediate **D** generates a four-coordinated Cu (III) complex **E**. Finally, the reductive elimination from **E** affords the desired dialkynylated intermediate **F**. Cu(I)I could coordinate with the electron-rich alkynyl bond of species **F** to give intermediate **G**. Afterwards, the *o*-alkynyl imidazoate salt **G** underwent intramolecular ring closure via oxyanion attacks at the C-β position of the acetylenic bond via 6-*endo*-*dig* cyclization to give intermediate **H**, which is further converted into **5** upon protodemetalation.

### Theoretical Calculation (Computational Studies)

**Figure 3:**
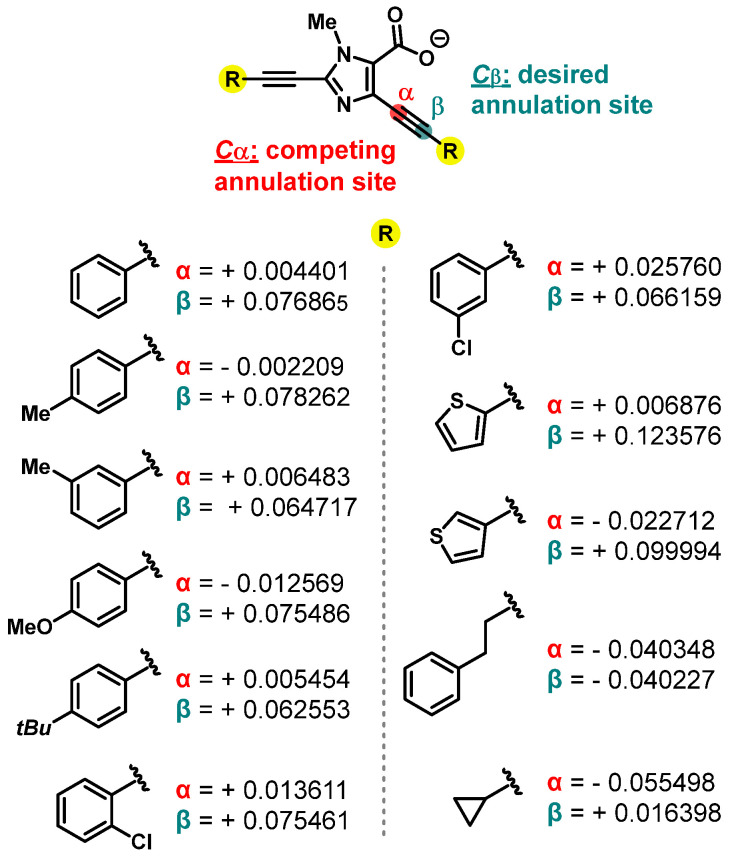
Plausible natural population analysis charge on the carbons Cα and Cβ of the alkyne substituent.

To validate our mechanistic hypothesis for intramolecular 6-*endo*-*dig* oxacyclization through β-addition, we conducted density functional theory (DFT) calculations to analyze the natural population of each carbon in the alkyne moiety, which appears to be critical in determining the resulting cyclization (Figure 3 and Supplementary Materials) [27,28,29,30,31,32,33].

The calculated NPA revealed that the partial positive charge is located on the β-carbon of alkyne leading to the 6-*endo*-*dig* oxacyclization, which is in excellent agreement with the experiment results, regardless of the nature of the alkyne substituent.

## 3. Materials and Methods

### 3.1. General Information

All reactions were performed under an inert atmosphere of argon in oven-dried glassware equipped with a magnetic stir bar. Solvents for reactions were obtained from Thermo Fisher Scientific (Illkirch-Graffenstaden, France) in extra dry quality and stored under argon over activated 3 Å sieves. All reagents were purchased from Fluorochem (Cork, Ireland) and used as received without additional purification. Reactions were monitored by thin-layer chromatography (TLC) analysis using silica gel 60 F254 plates. Column chromatography was performed using silica gel 60 (230–400.13 mesh, 0.040–0.063 mm). Eluents were distilled by the standard methods before each use. All new compounds were characterized by NMR spectroscopy (^1^H, and ^13^C), and high-resolution mass spectroscopy (HRMS). NMR spectra were recorded at 300 MHz for ^1^H, and 75 MHz for ^13^C with a Bruker^®^ (Wissembourg, France) 300 MHz NMR spectrometer. Proton and carbon magnetic resonance spectra (^1^H NMR and ^13^C NMR) were recorded using tetramethylsilane (TMS) as an external standard and CDCl_3_ (7.28 ppm for ^1^H NMR and 77.04 ppm for ^13^C NMR). Data for NMR are reported as follows: chemical shift (*δ* ppm), multiplicity (s = singlet, d = doublet, t = triplet, q = quartet, sep = septet, m = multiplet, and br = broad resonance), and coupling constants *J* are reported in Hertz (Hz). All NMR spectra were processed in MestReNova. HRMS experiments were performed on a hybrid tandem quadrupole/time-of-flight (Q-TOF) instrument, equipped with a pneumatically assisted electrospray (Z-spray) ion source (Micromass, Manchester, UK) operated in the positive mode. The melting points (Mp [°C]) of samples were measured using open capillary tubes and recorded on a Stuart^TM^ (Paris, France) melting point apparatus SMP3.

*Ethyl-2,4-diiodo-1H-imidazol-5-carboxylate* (**2**)

Compound **2** was prepared following a slightly modified procedure based on literature methods. Ethyl-1*H*-imidazole-5-carboxylate **1** (1.0 g, 7.1 mmol) was dissolved in dry acetonitrile (50 mL), and *N*-iodosuccinimide (NIS) (4.01 g, 17.8 mmol, 2.5 equiv.) was added to the solution. The reaction mixture was stirred at 80 °C for 3 h under an argon atmosphere. The progress of the reaction was monitored by TLC. Upon completion, the solvent was removed under reduced pressure. ^1^H NMR (300 MHz, CDCl_3_): *δ* = 8.28 (s, 1H), 4.40 (q, *J* = 7.1 Hz, 2H), 1.41 (t, *J* = 7.1 Hz, 3H). ^13^C NMR (75 MHz, CDCl_3_): *δ* = 177.6, 158.9, 91.2, 87.6, 59.7, 14.2.

*Ethyl-2,4-diiodo-1-methyl-1H-imidazol-5-carboxylate* (**3**)

Compound **3** was synthesized from ethyl-2,4-diiodo-1*H*-imidazole-5-carboxylate **2** (1.0 g, 2.55 mmol) and potassium *tert*-butoxide (0.31 g, 2.80 mmol), which were dissolved in dry DMF in a round-bottom flask under anhydrous conditions. The reaction mixture was cooled to 0 °C, and iodomethane was added dropwise. Stirring was continued for 24 h at room temperature under an argon atmosphere. After completion of the reaction (monitored by TLC), the solvent was removed under reduced pressure. The crude product was purified directly by column chromatography on silica gel using a mixture of petroleum ether and ethyl acetate as eluent, affording the desired product **3**. ^1^H NMR (300 MHz, CDCl_3_): *δ* = 4.37 (q, *J* = 7.1 Hz, 2H), 3.93 (s, 3H), 1.43 (t, *J* = 7.1 Hz, 3H). ^13^C NMR (75 MHz, CDCl_3_): *δ* = 177.6, 158.9, 89.3, 61.8, 29.6, 14.2. HRMS (ESI): *m*/*z* calcd for C_7_H_9_I_2_N_2_O_2_ [M + H]^+^ 406.8747; found: 406.8741.

*2,4-Diiodo-1-methyl-1H-imidazole-5-carboxylic acid* (**4**)

Compound **4** was obtained from ethyl-2,4-diiodo-1-methyl-1H-imidazole-5-carboxylate 3 (1.0 g, 2.45 mmol) and sodium hydroxide (0.14 g, 3.68 mmol), which were added to a round-bottom flask and dissolved in ethanol. The reaction mixture was stirred at 80 °C for 1 h. After completion of the reaction (monitored by TLC), the solvent was removed under reduced pressure. The residue was diluted with water, cooled to 0 °C, and acidified by the slow addition of 2 M HCl until the pH reached 1. The resulting precipitate was collected by vacuum filtration to afford compound **4**. ^1^H NMR (300 MHz, (CD_3_)_2_SO)): *δ* = 3.82 (s, 1H). ^13^C NMR (75 MHz, (CD_3_)_2_SO)): *δ* = 150.9, 122.7, 94.8, 78.6, 40.6, 40.3, 40.1, 39.8, 39.5, 39.2, 38.9, 30.6. HRMS (ESI): *m*/*z* calcd for C_5_H_5_I_2_N_2_O_2_ [M + H]^+^ 378.8434; found: 378.8429.

### 3.2. General Procedure for the Synthesis of 3-Methyl-6-aryl-2-alcynylpyrano[3,4-d]imidazole-4(3H)-one (**5a**–**k**)

An oven dried 25 mL Schlenk tube equipped with a magnetic stir bar was charged with 2,4-diiodo-1-methyl-1*H*-imidazole-5-carboxylic acid **5** (100 mg, 1 equiv.), CuI (2 equiv.), and K_2_CO_3_ (2 equiv.) solubilized in 4 mL of dry DMF. The vial was sealed with a septum-lined pierceable cap, evacuated, and back-filled with argon (×3). The terminal alkyne (3 equiv.) was dissolved in anhydrous DMF (2 mL) and added to the reaction mixture via a syringe. The reaction vessel was placed in a pre-heated oil bath and stirred at 130 °C for 3 h under an argon atmosphere. After completion of the reaction (monitored by TLC), the mixture was cooled to room temperature and filtered through a thin pad of celite eluting with ethyl acetate (25 mL), and the combined filtrate was concentrated in vacuo. The residue was directly purified by column chromatography on silica gel using a mixture of petroleum ether/EtOAc as eluent to give the desired products **5a**–**k**.

*3-Methyl-6-phenyl-2-(phenylethynyl)pyrano[3,4-d]imidazol-4(3H)-one* (**5a**)

The crude product was purified by column chromatography on silica gel (PE/EtOAC, 9.5:0.5), followed by recrystallization from Et_2_O to afford compound **5a** as a white solid (43.8 mg, 51%). Mp 223–225 °C. ^1^H NMR (300 MHz, CDCl_3_): *δ* = 7.87–7.84 (m, 2H), 7.66–7.65 (m, 2H), 7.47–7.42 (m, 6H), 7.12 (s, 1H), 4.16 (s, 3H) ppm. ^13^C NMR (75 MHz, CDCl_3_): *δ* = 156.6, 155.1, 150.0, 140.3, 132.2, 132.0, 130.3, 129.9, 128.9, 128.7, 125.4, 120.5, 117.3, 98.3, 97.2, 77.3, 33.2. HRMS (ESI): *m*/*z* calcd for C_21_H_15_N_2_O_2_ [M + H]^+^ 327.1128; found: 327.1120.

*3-Methyl-6-(4-methylphenyl)-2-[(4-methylphenyl)ethynyl]pyrano[3,4-d]imidazol-4(3H)-one* (**5b**)

The crude product was purified by column chromatography on silica gel (PE/EtOAC, 9.5:0.5), followed by recrystallization from Et_2_O to afford compound **5b** as a white solid (65.1 mg, 70%). Mp 179–181 °C. ^1^H NMR (300 MHz, CDCl_3_): *δ* = 7.78–7.75 (d, *J* = 8.2 Hz, 2H), 7.56–7.53 (d, *J* = 8.1 Hz, 2H), 7.29–7.23 (m, 4H), 7.08 (s, 1H), 4.17 (s,3H), 2.43 (s, 6H) ppm. ^13^C NMR (75 MHz, CDCl_3_): *δ* = 156.5, 155.0, 150.0, 140.2, 132.2, 132.0, 130.2**,** 129.9, 128.8, 128.7, 125.3, 120.4, 117.2, 98.2, 97.2, 77.2, 33.2. HRMS (ESI): *m*/*z* calcd for C_23_H_19_N_2_O_2_ [M + H]^+^ 355.1441; found: 355.1433.

*3-Methyl-6-(3-methylphenyl)-2-[(3-methylphenyl)ethynyl]pyrano[3,4-d]imidazol-4(3H)-one* (**5c**)

The crude product was purified by column chromatography on silica gel (PE/EtOAC, 9.5:0.5), followed by recrystallization from Et_2_O to afford compound **5c** as a white solid (63.7 mg, 68%). Mp 186–188 °C. ^1^H NMR (300 MHz, CDCl_3_): *δ* = 7.68–7.62 (m, 2H), 7.45–7.43 (m, 2H), 7.37–7.22 (m, 4H), 7.09 (s, 1H), 4.15 (s,3H), 2.42 (s, 3H), 2.39 (s, 3H) ppm. ^13^C NMR (75 MHz, CDCl_3_): *δ* = 156.8, 155.1, 150.1, 140.3, 138.6, 138.5, 132.7**,** 132.0, 131.2, 130.7, 129.3, 128.7, 128.6, 126.0, 122.5, 120.3, 117.2, 98.2, 97.5, 33.2, 21.5, 21.2. HRMS (ESI): *m*/*z* calcd for C_23_H_19_N_2_O_2_ [M + H]^+^ 355.1441; found: 355.1434.

*6-(4-Methoxyphenyl)-2-[(4-methoxyphenyl)ethynyl]-3-methylpyrano[3,4-d]imidazol-4(3H)-one* (**5d**)

The crude product was purified by column chromatography on silica gel (PE/EtOAC, 9.5:0.5), followed by recrystallization from Et_2_O to afford compound **5d** as a white solid (79.5 mg, 78%). Mp 253–255 °C. ^1^H NMR (300 MHz, CDCl_3_): *δ* = 7.81–7.78 (d, *J* = 8.8 Hz, 2H), 7.59–7.56 (d, *J* = 8.7 Hz, 2H), 6.99–6.94 (m, 4H), 6.91 (s, 1H), 4.14 (s,3H), 3.86 (s, 6H) ppm. ^13^C NMR (75 MHz, CDCl_3_): *δ* = 161.1, 161.0, 156.6, 155.2, 150.4, 140.6, 133.9**,** 133.2, 126.9, 124.7, 116.6, 114.4, 114.2, 112.4, 97.6, 96.7, 76.5, 55.4, 33.1. HRMS (ESI): *m*/*z* calcd for C_23_H_19_N_2_O_4_ [M + H]^+^ 387.1339; found: 387.1332.

*6-(4-(Tert-butyl)phenyl)-2-((4-(tert-butyl)phenyl)ethynyl)-3-methylpyrano[3,4-d]imidazol-4(3H)-one* (**5e**)

The crude product was purified by column chromatography on silica gel (PE/EtOAC, 9.5:0.5), followed by recrystallization from Et_2_O to afford compound **5e** as a white solid (27.8 mg, 44%). Mp 261–263 °C. ^1^H NMR (300 MHz, CDCl_3_): *δ* = 7.80 (d, *J* = 8.6 Hz, 2H), 7.58 (d, *J* = 8.4 Hz, 2H), 7.49–7.43 (m, 4H), 7.12 (s, 1H), 4.16 (s,3H), 1.35 (s, 9H), 1.34 (s, 9H) ppm. ^13^C NMR (75 MHz, CDCl_3_): *δ* = 161.1, 161.0, 156.6, 155.2, 150.4, 140.6, 133.9, 133.2, 126.9, 124.7, 116.6, 114.4, 114.2, 112.4, 97.6, 96.7, 76.5, 55.4, 33.1. HRMS (ESI): *m*/*z* calcd for C_29_H_31_N_2_O_2_: 439.2380; found: 439.2376.

*6-(2-Chlorophenyl)-2-[(2-chlorophenyl)ethynyl]-3-methylpyrano[3,4-d]imidazol-4(3H)-one* (**5f**)

The crude product was purified by column chromatography on silica gel (PE/EtOAC, 9.5:0.5), followed by recrystallization from Et_2_O to afford compound **5f** as a white solid (74.8 mg, 58%). Mp 179–181 °C. ^1^H NMR (300 MHz, CDCl_3_): *δ* = 7.70–7.66 (m, 2H), 7.51–7.48 (m, 2H), 7.41–7.33 (m, 4H), 7.15 (s, 1H), 4.22 (s,3H) ppm. ^13^C NMR (75 MHz, CDCl_3_): *δ* = 155.3, 154.5, 149.6, 140.1, 136.7, 134.1, 132.6, 131.8, 131.4, 130.9, 130.9, 130.8, 129.8, 127.1, 127.0, 120.9, 117.8, 104.2, 93.8, 82.1, 33.6. HRMS (ESI): *m*/*z* calcd for C_21_H_13_Cl_2_N_2_O_2_: 395.0348; found: 395.0342.

*6-(3-Chlorophenyl)-2-[(3-chlorophenyl)ethynyl]-3-methylpyrano[3,4-d]imidazol-4(3H)-one* (**5g**)

The crude product was purified by column chromatography on silica gel (PE/EtOAC, 9.5:0.5), followed by recrystallization from Et_2_O to afford compound **5e** as a white solid (22.7 mg, 65%). Mp 225–227 °C. ^1^H NMR (300 MHz, CDCl_3_): *δ* = 7.85–7.52 (m, 4H), 7.47–7.34 (m, 4H), 7.12 (s, 1H), 4.17 (s,3H) ppm. ^13^C NMR (75 MHz, CDCl_3_): *δ* = 155.2, 154.7, 149.8, 140.1, 135.3, 134.8, 133.8, 132.1, 130.8, 130.5, 130.3, 130.1, 130.1, 125.6, 123.5, 122.2, 117.8, 99.2, 95.7, 78.2, 33.5. HRMS (ESI): *m*/*z* calcd for C_21_H_13_Cl_2_N_2_O_2_: 395.0348; found: 395.0342.

*3-Methyl-6-(thiophen-2-yl)-2-(thiophen-2-ylethynyl)pyrano[3,4-d]imidazol-4(3H)-one* (**5h**)

The crude product was purified by column chromatography on silica gel (PE/EtOAC, 9.5:0.5), followed by recrystallization from Et_2_O to afford compound **5e** as a white solid (40 mg, 55%). Mp 197–199 °C. ^1^H NMR (300 MHz, CDCl_3_): *δ* = 7.85–7.74 (m, 2H), 7.43–7.36 (m, 3H), 7.29–7.27 (m, 1H), 6.94 (s, 1H), 4.13 (s, 3H) ppm. ^13^C NMR (75 MHz, CDCl_3_): *δ* = 155.0, 153.5, 150.1, 140.4, 134.4, 132.2, 129.9, 127.1, 126.4, 124.6, 124.2, 119.7, 117.1, 97.9, 92.8, 77.2, 33.4. HRMS (ESI): *m*/*z* calcd for C_17_H_12_N_2_O_2_S_2_: 339.0256; found: 339.0249.

*3-Methyl-6-(thiophen-3-yl)-2-(thiophen-3-ylethynyl)pyrano[3,4-d]imidazol-4(3H)-one* (**5i**)

The crude product was purified by column chromatography on silica gel (PE/EtOAC, 9.5:0.5), followed by recrystallization from Et_2_O to afford compound **5e** as a white solid (22.2 mg, 41%). Mp 218–220 °C. ^1^H NMR (300 MHz, CDCl_3_): *δ* = 7.85 (dd, *J* = 3.0, 1.4 Hz, 1H), 7.75 (dd, *J* = 3.0, 1.1 Hz, 1H), 7.42 (dd, *J* = 5.1, 1.4 Hz, 1H), 7.40–7.37 (m, 2H), 7.29 (dd, *J* = 5.0, 1.2 Hz, 1H), 6.94 (s, 1H), 4.13 (s, 3H) ppm. ^13^C NMR (75 MHz, CDCl_3_): *δ* = 155.1, 153.4, 150.2, 140.4, 134.4, 132.2, 129.9, 127.1, 126.4, 124.6, 124.2, 119.7, 117.1, 98.0, 92.6, 77.2, 33.4. HRMS (ESI): *m*/*z* calcd for C_17_H_11_N_2_O_2_S_2_: 339.0256; found: 339.0252.

*3-Methyl-6-phenethyl-2-(4-phenylbut-1-yn-1-yl)pyrano[3,4-d]imidazol-4(3H)-one* (**5j**)

The crude product was purified by column chromatography on silica gel (PE/EtOAC, 9.5:0.5), followed by recrystallization from Et_2_O to afford compound **5e** as a white solid (28.1 mg, 49%). Mp 165–167 °C. ^1^H NMR (300 MHz, CDCl_3_): *δ* = 7.29–7.21 (m, 8H), 7.21–7.19 (m, 2H), 6.35 (s, 1H), 3.85 (s, 3H), 3.00–2.96 (m, 4H), 2.86–2.82 (m, 4H) ppm. ^13^C NMR (75 MHz, CDCl_3_): *δ* = 159.8, 155.9, 149.6, 140.2, 140.0, 139.7, 128.7, 128.6, 128.5, 128.4, 126.8, 126.4, 116.2, 100.3, 98.7, 70.2, 35.6, 34.1, 33.5, 32.9, 29.8, 21.6. HRMS (ESI): *m*/*z* calcd for C_25_H_23_N_2_O_2_: 383.1754; found: 383.1749.

*6-Cyclopropyl-2-(cyclopropylethynyl)-3-methylpyrano[3,4-d]imidazol-4(3H)-one* (**5k**)

The crude product was purified by column chromatography on silica gel (PE/EtOAC, 9.5:0.5), followed by recrystallization from Et_2_O to afford compound **5e** as a white solid (21.4 mg, 52%). Mp 132–134 °C. ^1^H NMR (300 MHz, CDCl_3_): *δ* = 6.41 (s, 1H), 3.96 (s, 3H), 1.87–1.77 (m, 2H), 1.58–1.49 (m, 1H), 1.04–0.94 (m, 8H) ppm. ^13^C NMR (75 MHz, CDCl_3_): *δ* = 161.4, 155.6, 150.2, 140.4, 102.7, 98.0, 64.5, 33.0, 29.8, 14.2, 9.4, 7.5, 0.2. HRMS (ESI): *m*/*z* calcd for C_15_H_15_N_2_O_2_: 255.1128; found: 255.1123.

*Ethyl 1-methyl-2,4-bis(phenylethynyl)-1H-imidazole-5-carboxylate* (**6**)

Compound **6** was synthesized according to the General Procedure for the Synthesis of 3-methyl-6-aryl-2-alkynylpyrano[3,4-*d*]imidazole-4(3*H*)-ones (**5a**–**k**), described above. After purification by column chromatography on silica gel (PE/EtOAC, 9.5:0.5), compound **6** was obtained as a white solid (36 mg, 41%). ^1^H NMR (300 MHz, CDCl_3_): *δ* = 7.61–7.55 (m, 4H), 7.43–7.34 (m, 6H), 4.42 (q, *J* = 6.6 Hz, 1H), 4.05 (s, 1H), 1.45 (t, *J* = 6.8 Hz, 1H). ^13^C NMR (75 MHz, CDCl_3_): *δ* = 159.8, 136.2, 132.2, 131.9, 131.2, 129.9, 128.7, 128.5, 123.0, 121.1, 95.1, 93.0, 83.0, 61.3, 34.6, 14.5.

## 4. Conclusions

In conclusion, we have developed a novel approach for one-pot copper-mediated tandem Sonogashira coupling/regioselective 6-*endo*-*dig* oxacyclization reaction of 2,4-diiodo-1-methyl-1*H*-imidazole-5-carboxylic acid and terminal alkynes. The following unprecedented method demonstrates broad compatibility with structurally diverse al-iphatic, aromatic, and heteroaromatic alkynes. Notably, 5-exo-dig heterocycles were not detected in all experiments, demonstrating the high regioselectivity of the intramolecular 6-*endo*-*dig* oxacyclization step. Furthermore, theoretical calculations (computational results) indicated that the favorable reaction position is the β-carbon of alkyne, which is in close agreement with the experimental results.

## Figures and Tables

**Table 1:** Optimization of the reaction conditions between 2,4-diiodo-1-methyl-1*H*-imidazole-5-carboxylic acid **4** and phenylacetylene.

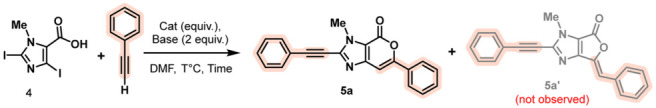						
Entry	Base	Catalyst (equiv.)	Alkyne (equiv.)	T (°C)	Time (h)	Ratio (%) ^a^5a/4
1	Et_3_N	CuI/PdCl_2_(PPh_3_)_2_ (0.2/0.1)	3	130	3	47/53
2	Et_3_N	CuI/PdCl_2_(PPh_3_)_2_ (1/0.1)	3	130	3	70/30
3	K_2_CO_3_	CuI/PdCl_2_(PPh_3_)_2_ (1/0.1)	3	130	3	100/0 (44%) ^b^
4	K_2_CO_3_	CuI (1)	3	130	24	100/0 (27%) ^b^
5	K_2_CO_3_	CuI (2)	3	130	3	100/0 (51%) ^b^
6	K_2_CO_3_	CuI (2.5)	3	130	3	100/0 (47%) ^b^
7	K_2_CO_3_	CuI (2)	2.05	130	3	100/0 (24%) ^b^
8	K_2_CO_3_	CuI (2)	1.05	130	3	100/0 (12%) ^b^
9	K_2_CO_3_	CuI (2)	3	150	3	100/0 (44%) ^b^
10	K_2_CO_3_	CuI (2)	3	100	24	100/0 (12%) ^b^
11	Na_2_CO_3_	CuI (2)	3	130	3	100/0 (9%) ^b^
12	K_3_PO_4_	CuI (2)	3	130	3	55/45
13	Cs_2_CO_3_	CuI (2)	3	130	3	52/48
14	Et_3_N	CuI (2)	3	130	3	55/45

^a^ The ratio of mixture (**5a**/**4**) was calculated from the crude ^1^H NMR spectrum. ^b^ Isolated yield.

## Data Availability

The data set presented in this study is available in this article.

## Supplementary Materials

The following supporting information can be downloaded at https://www.mdpi.com/article/10.3390/molecules30143045/s1, S1. Copies of ^1^H and ^13^C and NMR spectra, S2. HRMS spectra, S3. Details of DFT calculations, S4. References.
