# Supplementary material for: Pd/Ligand-Free Synthesis of 2-Alkynylated Pyrano[4,3-d]imidazol-4-ones via One-Pot Cu-Mediated Tandem Sonogashira Coupling/Regioselective 6-endo-dig Oxacyclization Reaction
Source: Molecules. 2025 Jul 21;30(14):3045. doi: 10.3390/molecules30143045 (PMC12300436; doi:10.3390/molecules30143045)
Supplement: Supplementary file 1 [file molecules-30-03045-s001.zip › molecules-3729228-supplementary.pdf]

Supporting information for

Pd/ligand-free synthesis of 2-alkynylated pyrano[4,3-*d*]imidazol-4-one *via* one-pot Cu-mediated Sonogashira coupling and Tandem Sonogashira coupling/regioselective 6-*endo-dig* oxacyclization reaction

Table of Contents

1. Copies of ^{1}H, ^{13}C and NMR spectra S1-S14

2. HRMS spectra S17-S23

3. Details of DFT calculations S23-S40

4. References S41

1. Copies of ^{1}H, ^{13}C and NMR spectra

^{1}H NMR (300 MHz, $CDCl_3$) **2**

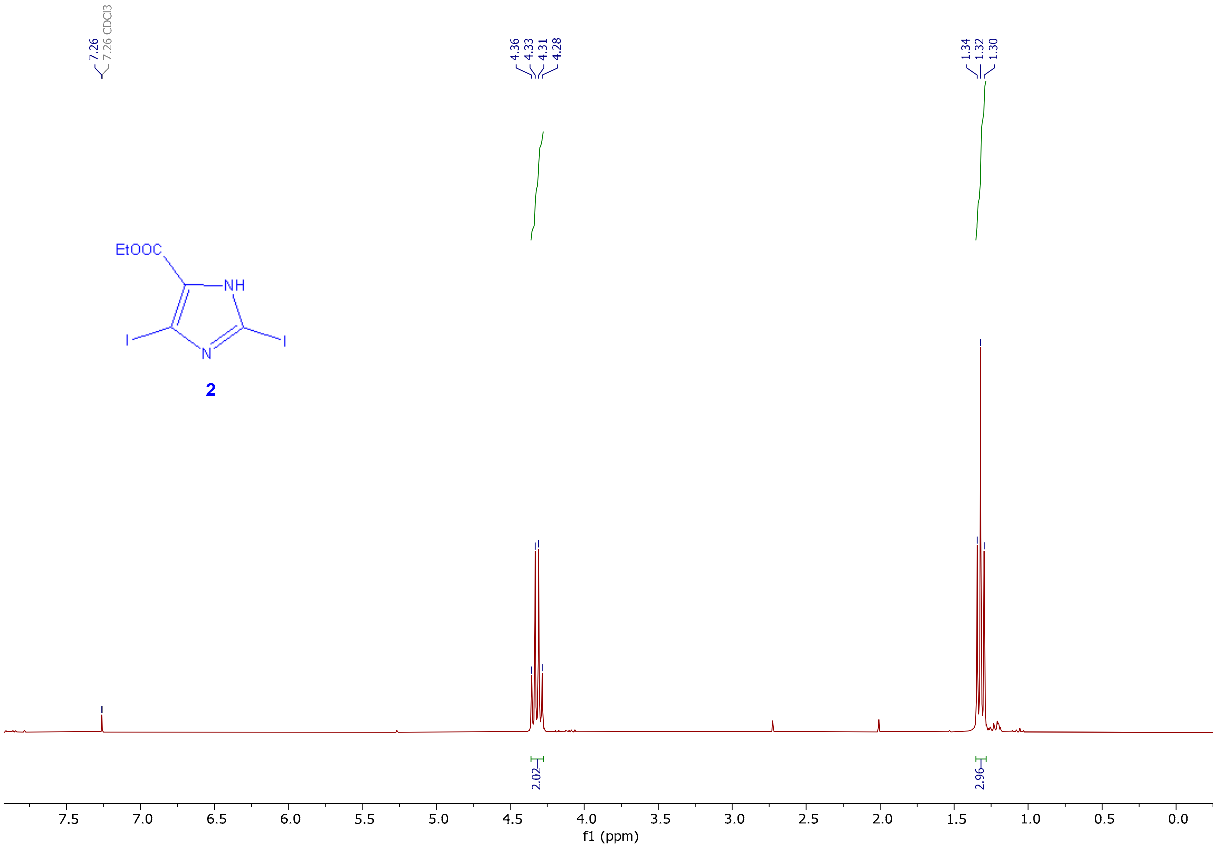

^{13}C NMR (75 MHz, $CDCl_3$) **2**

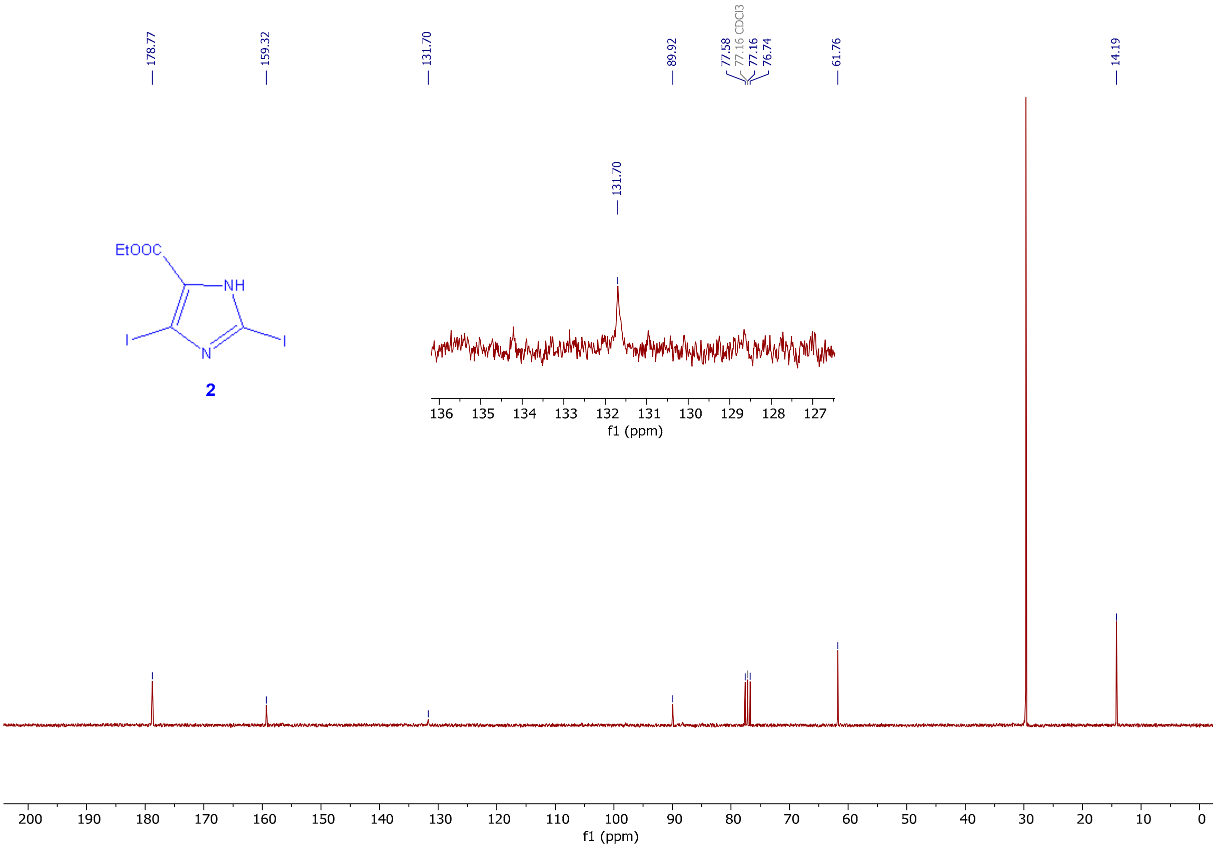

^{1}H NMR (300 MHz, $CDCl_3$) **3**

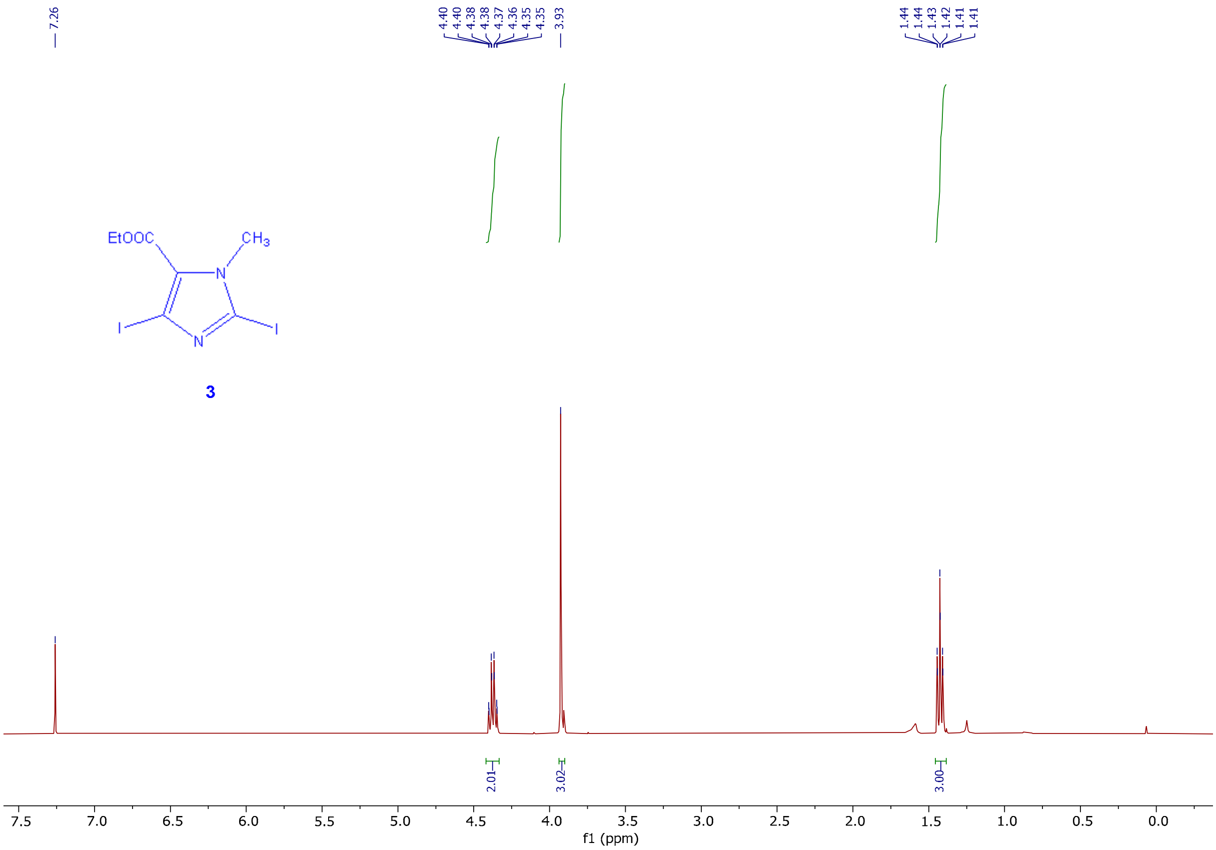

^{13}C NMR (75 MHz, $CDCl_3$) **3**

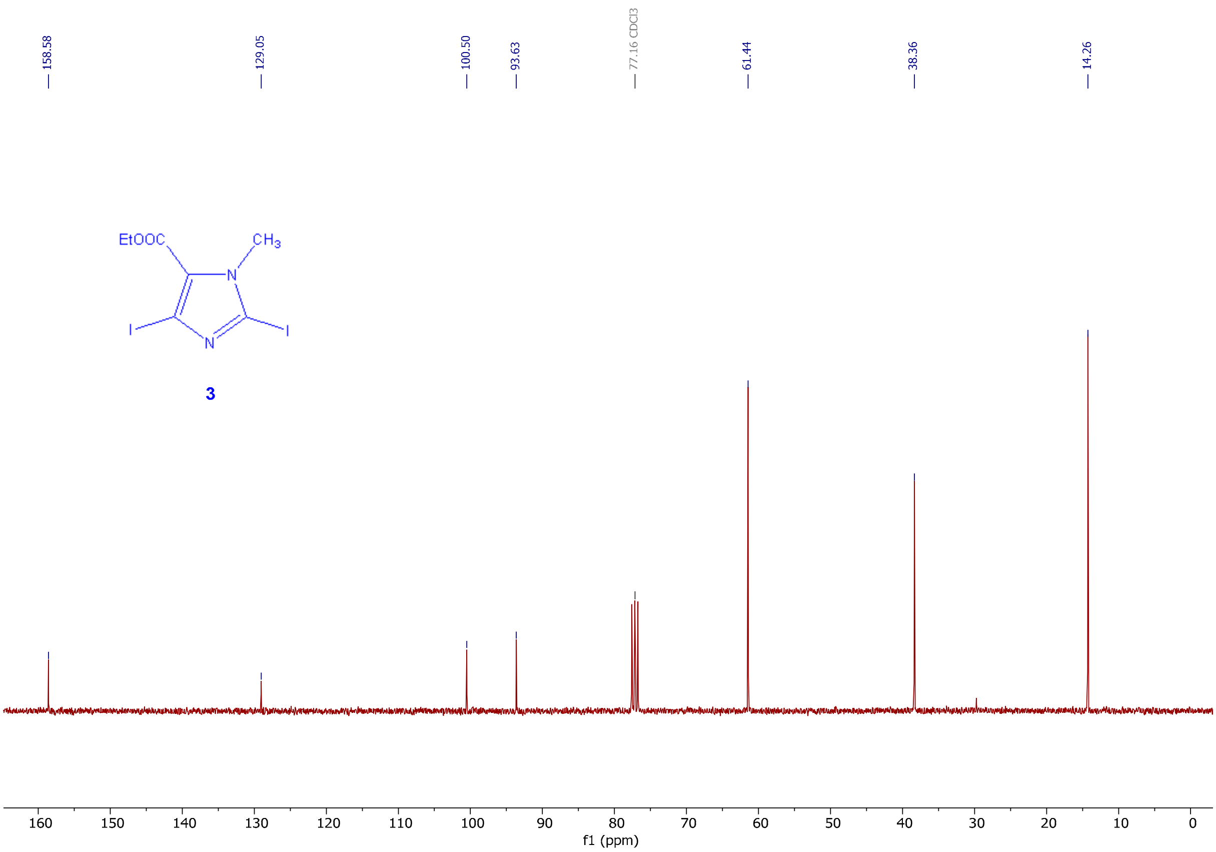

^{1}H NMR (300 MHz, $CDCl_3$) **4**

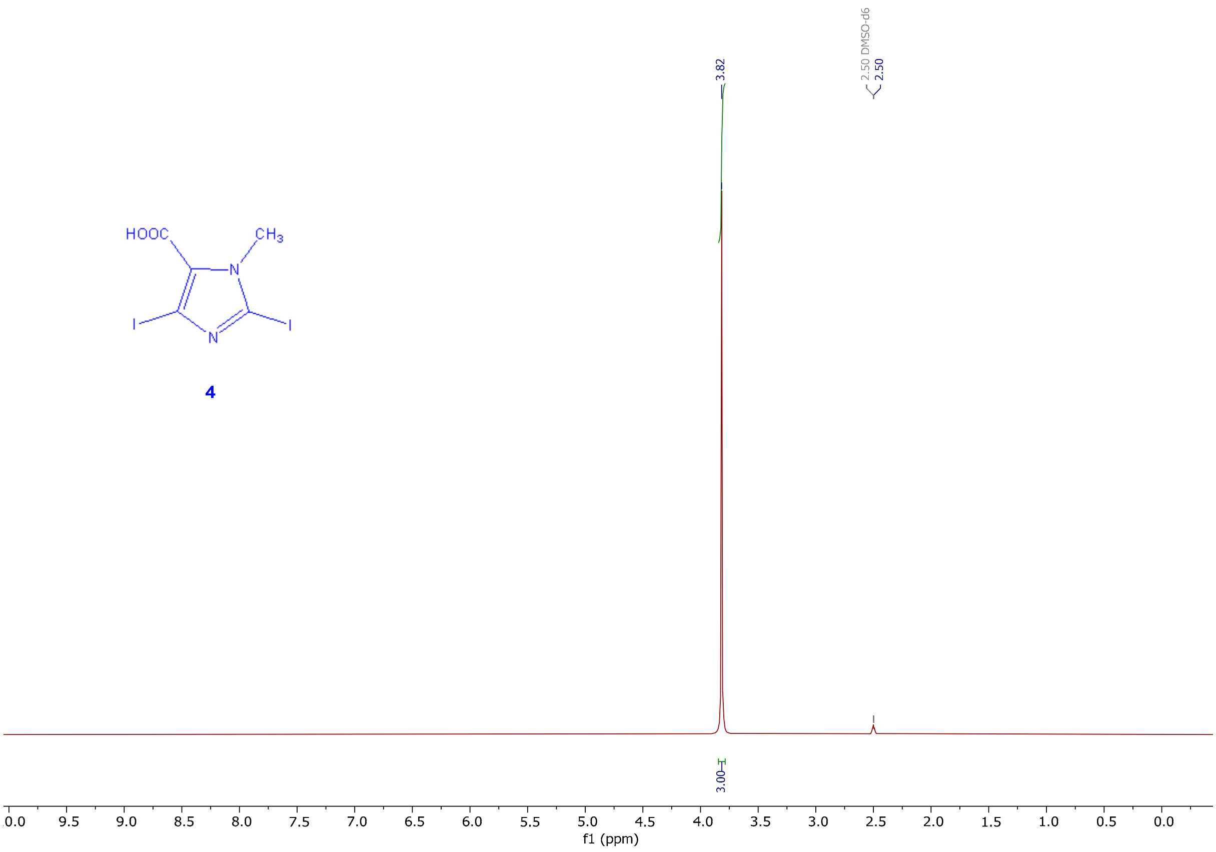

^{13}C NMR (75 MHz, $(CD_3)_2SO$) **4**

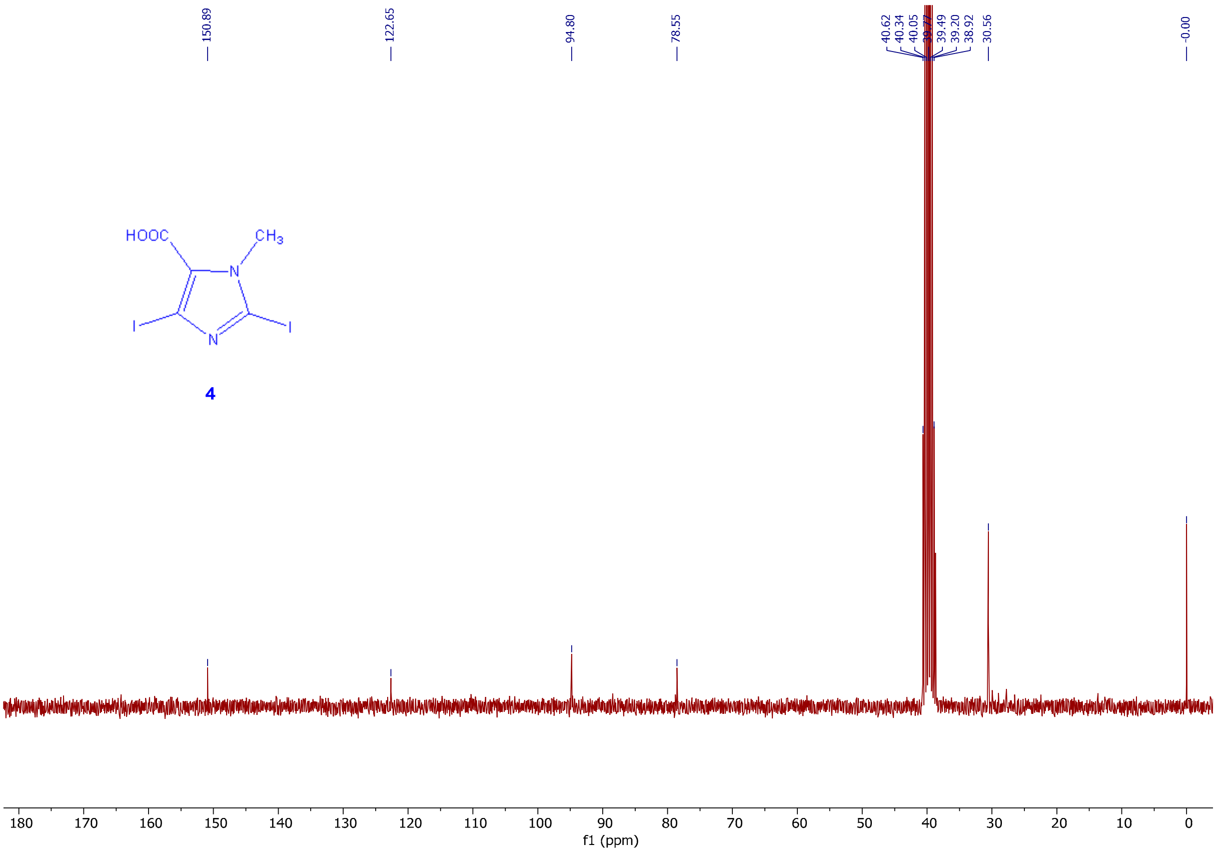

^{1}H NMR (300 MHz, $CDCl_3$) of **5a**

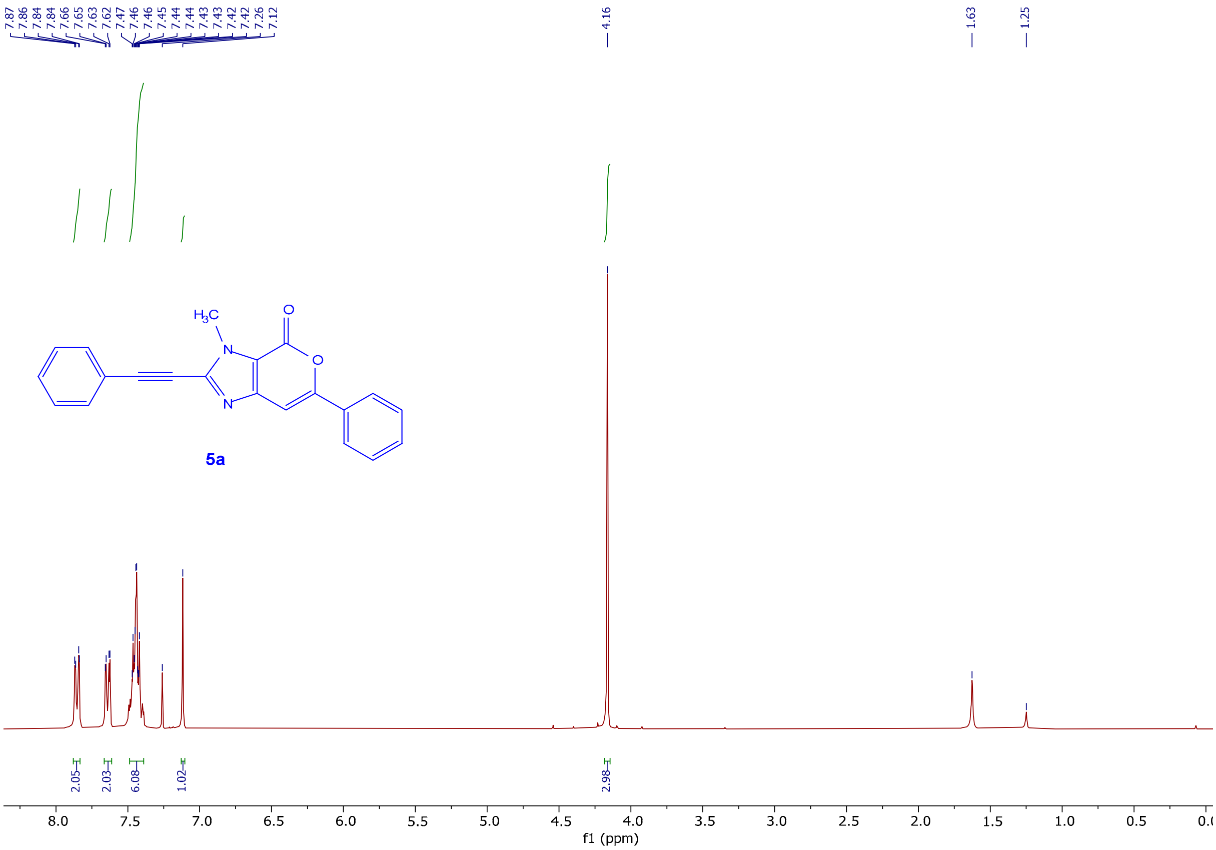

^{13}C NMR (75 MHz, $CDCl_3$) of **5a**

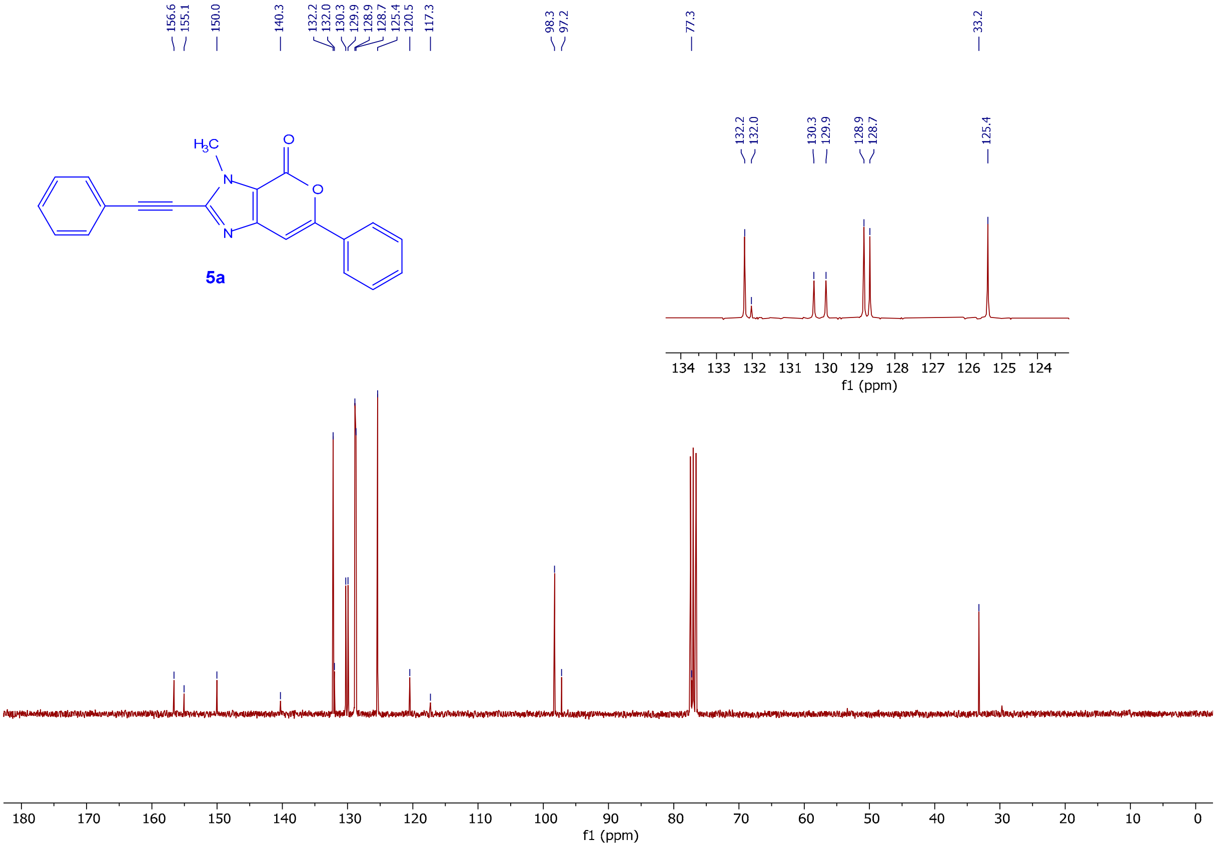

^{1}H NMR (300 MHz, $CDCl_3$) of **5b**

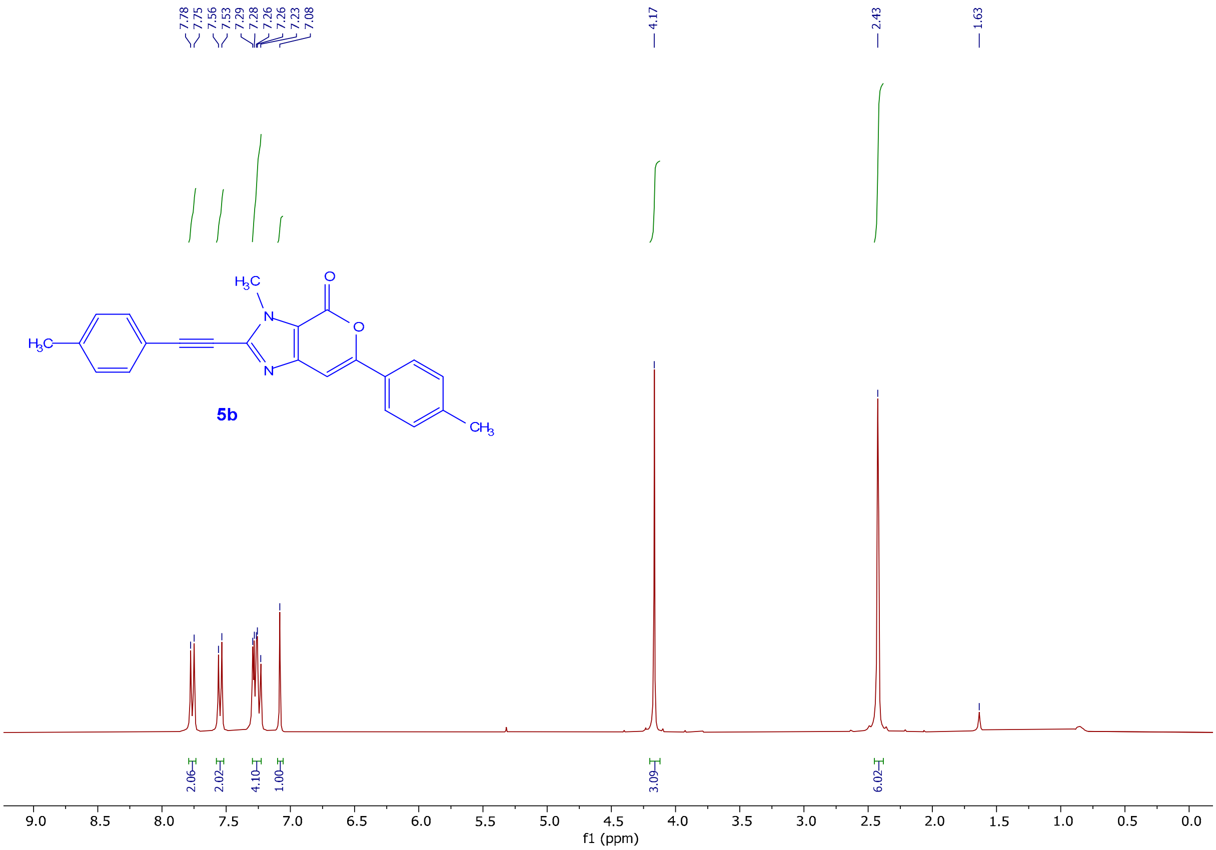

^{13}C NMR (75 MHz, $CDCl_3$) of **5b**

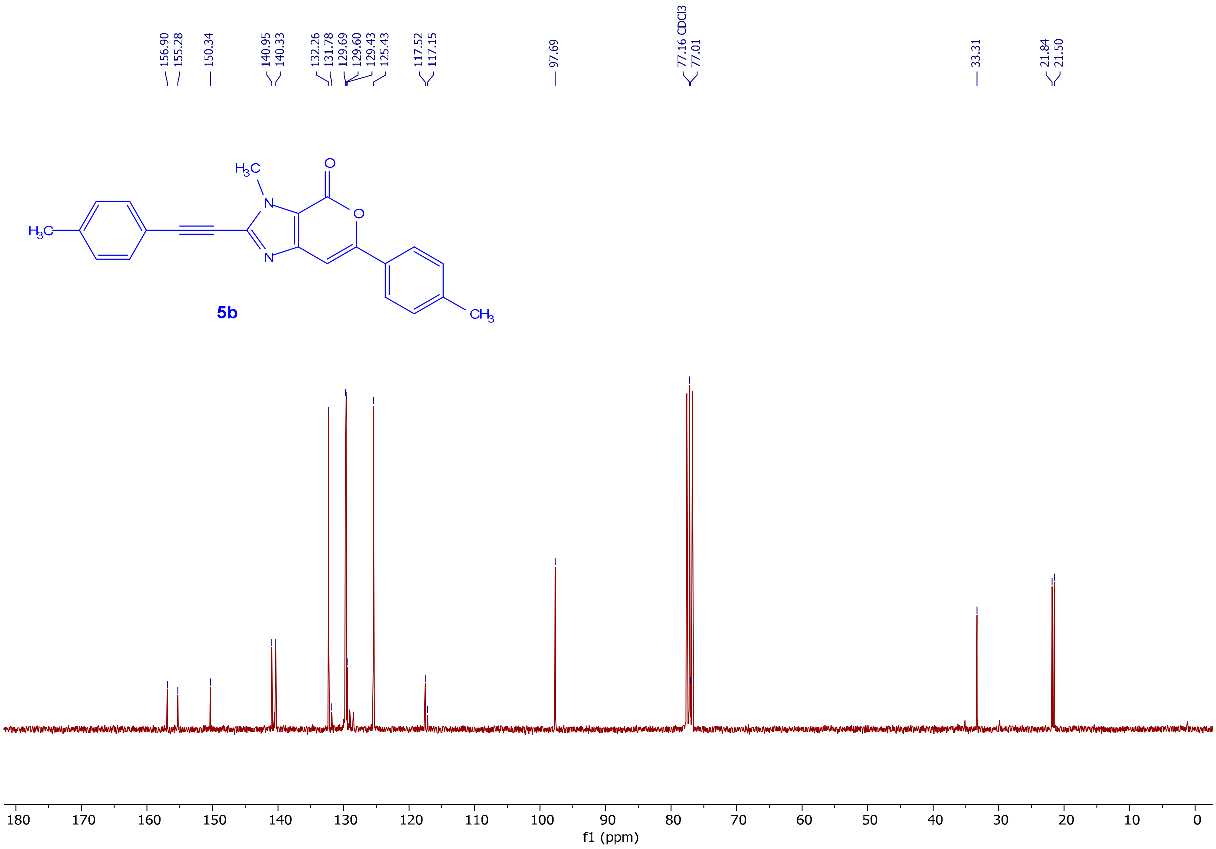

^{1}H NMR (300 MHz, $CDCl_3$) of **5c**

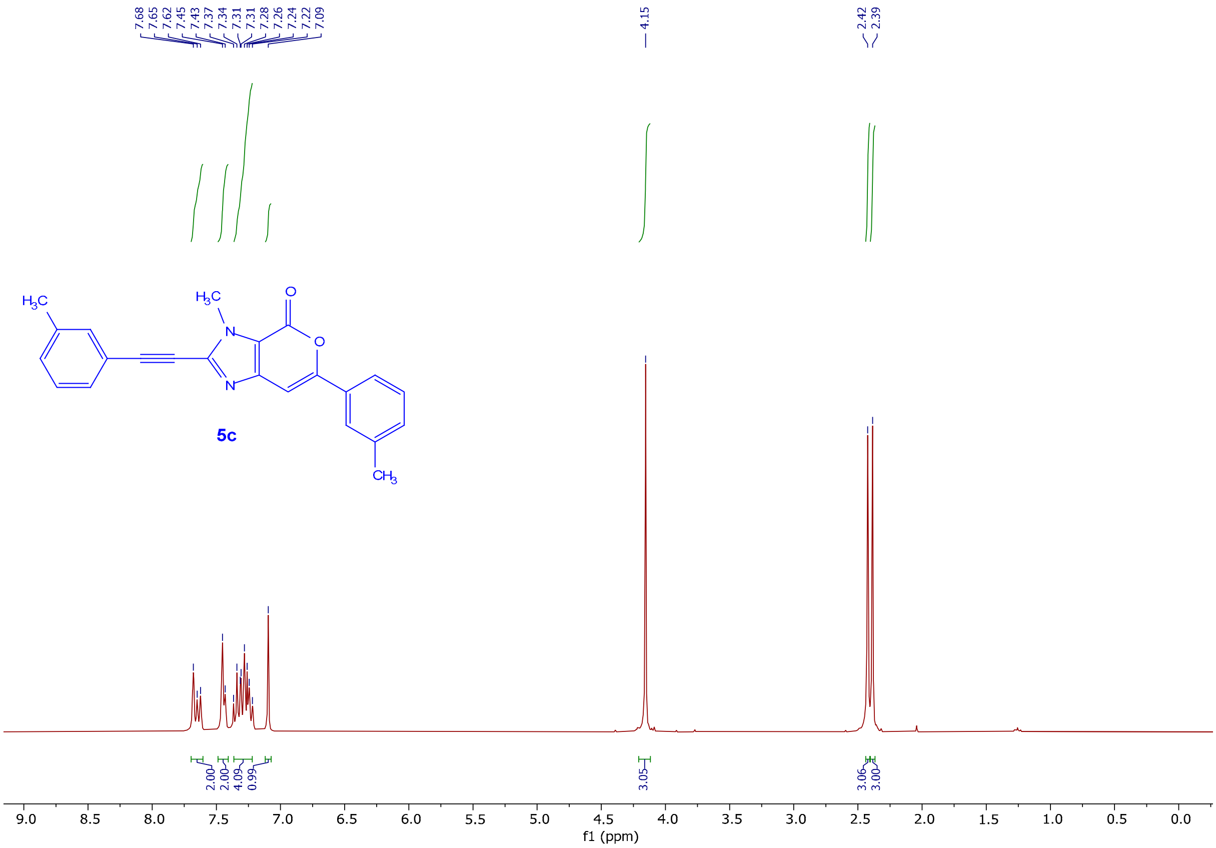

^{13}C NMR (75 MHz, $CDCl_3$) of **5c**

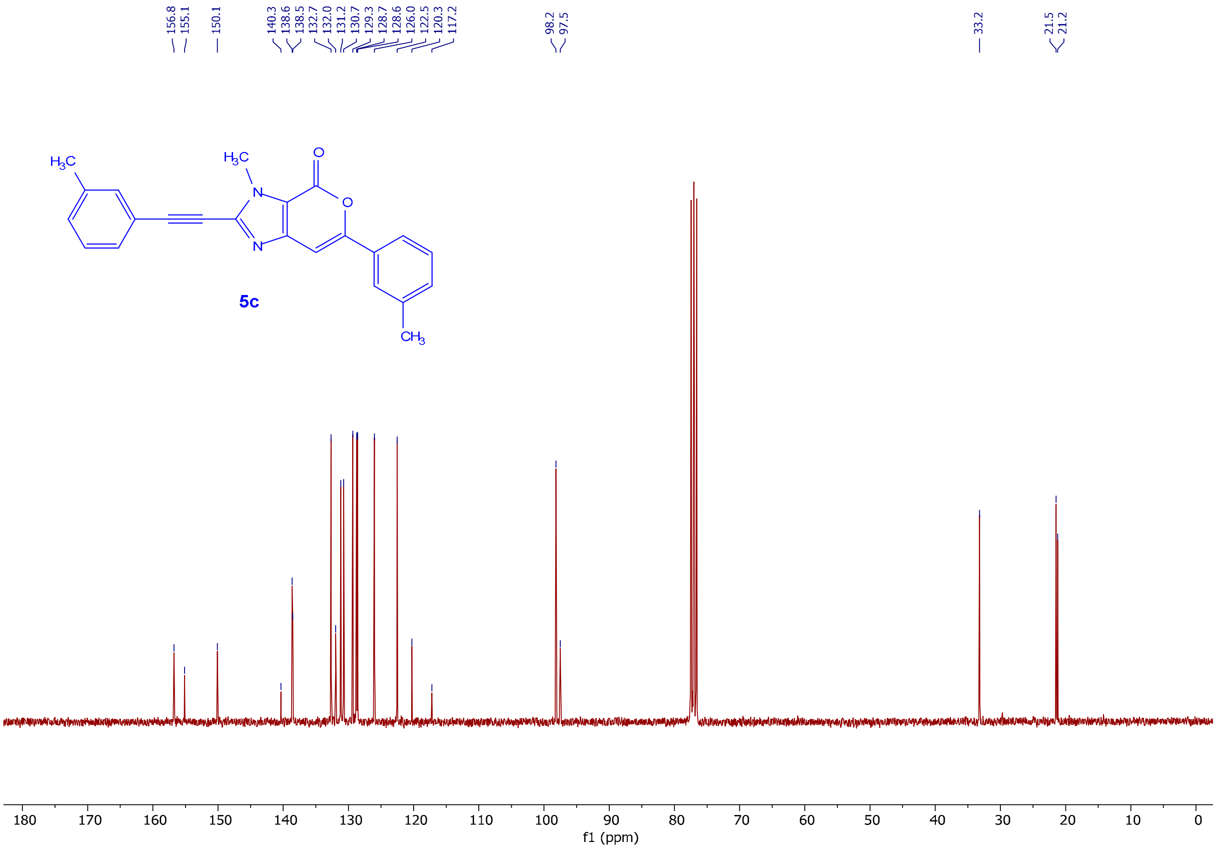

^{1}H NMR (300 MHz, $CDCl_3$) of **5d**

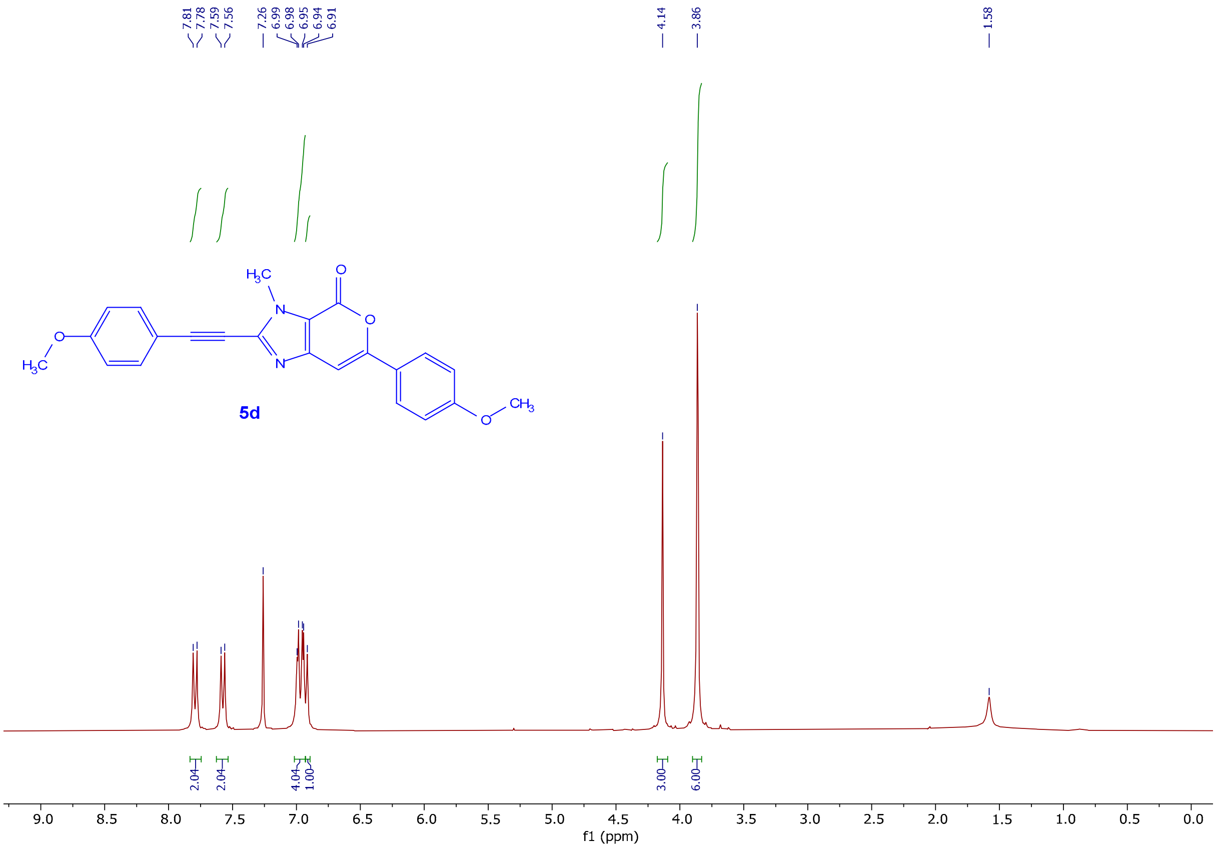

^{13}C NMR (75 MHz, $CDCl_3$) of **5d**

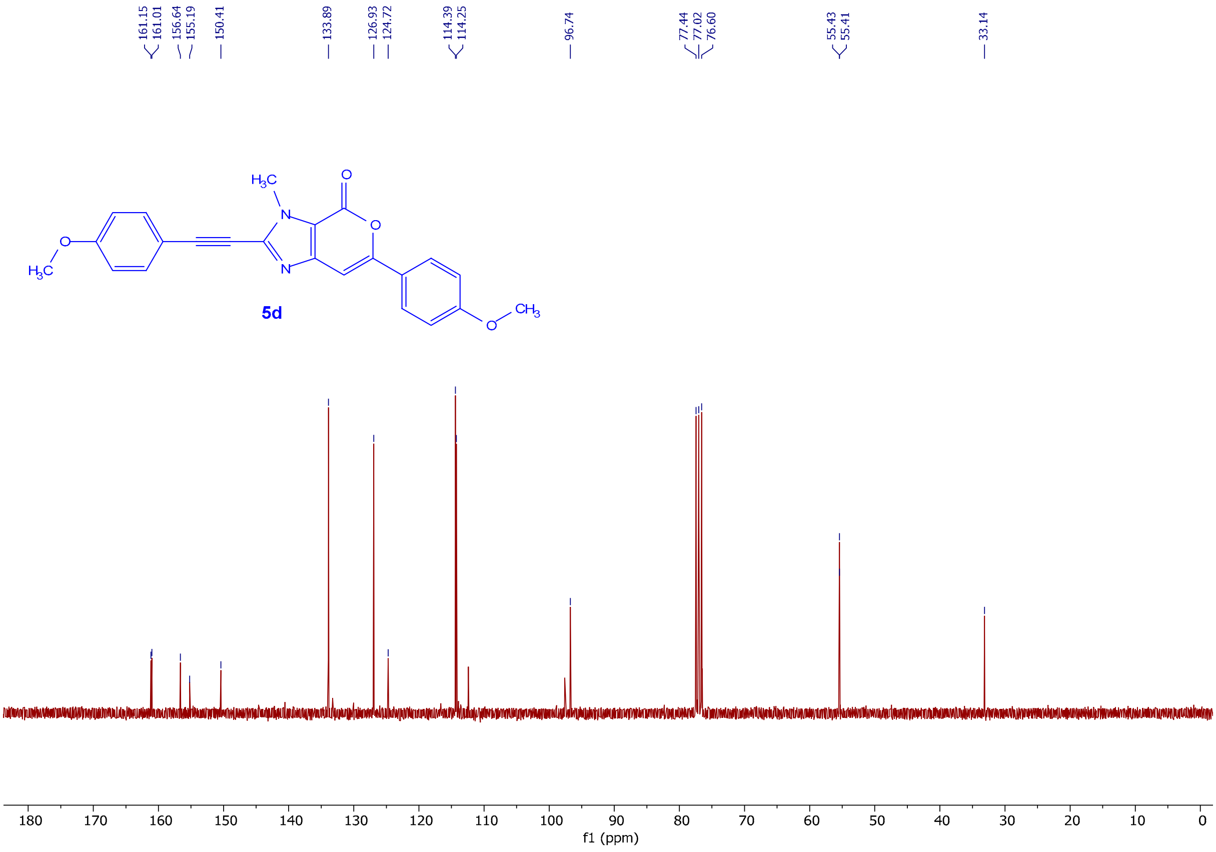

^{1}H NMR (300 MHz, $CDCl_3$) of **5e**

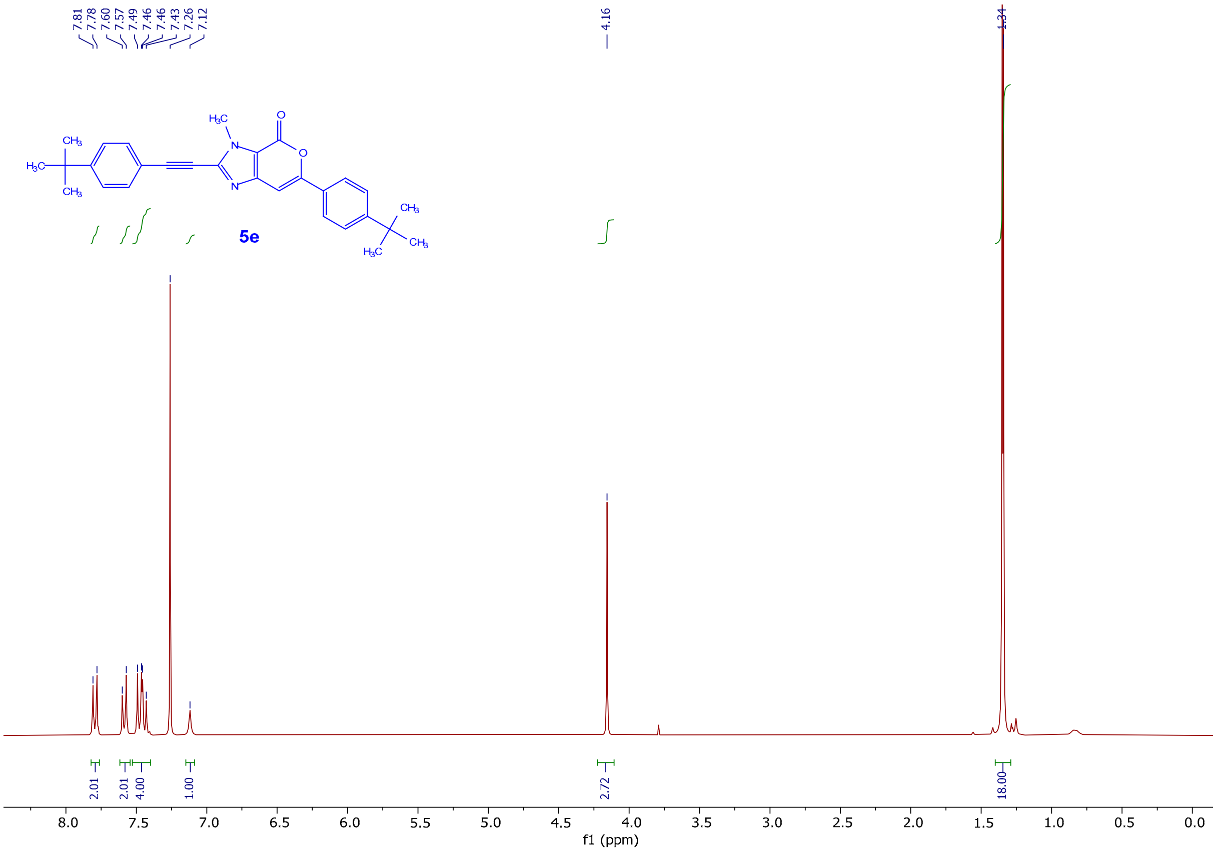

^{13}C NMR (75 MHz, $CDCl_3$) of **5e**

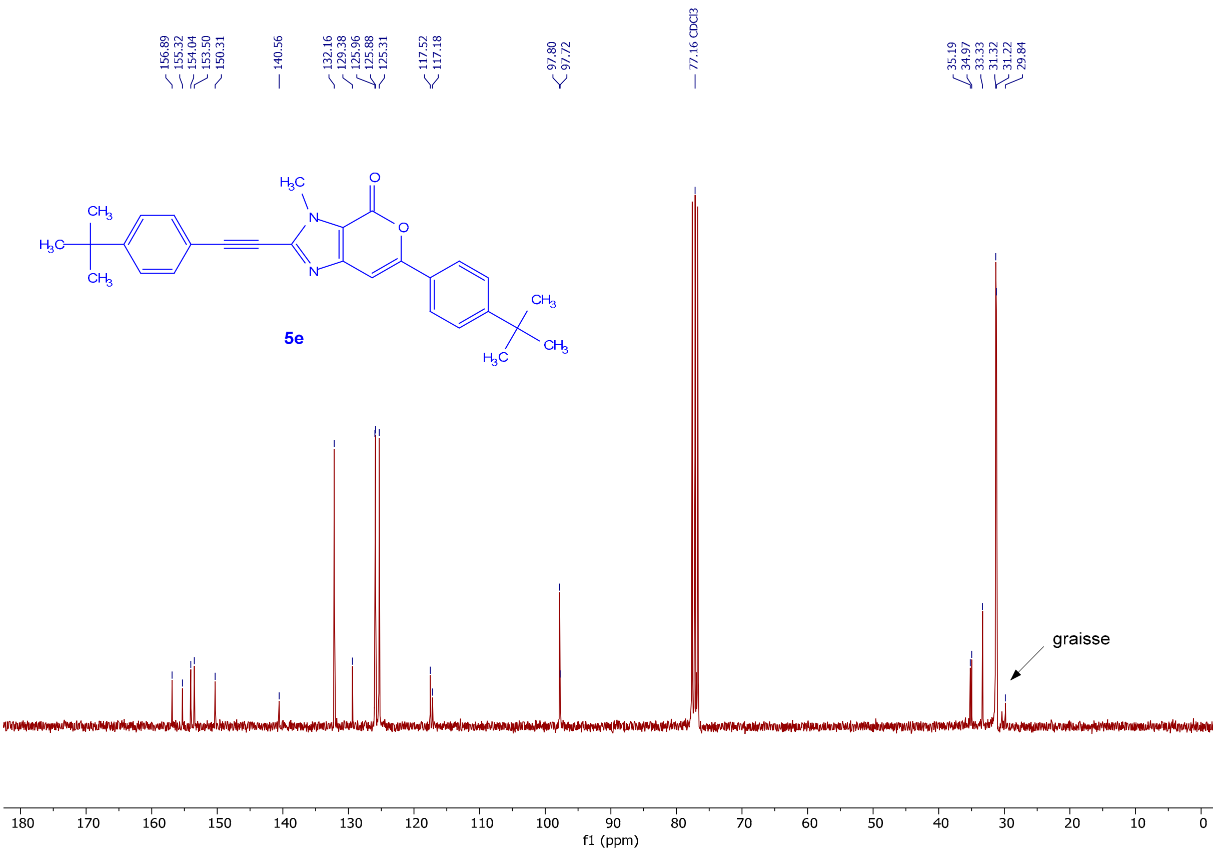

^{1}H NMR (300 MHz, $CDCl_3$) of **5f**

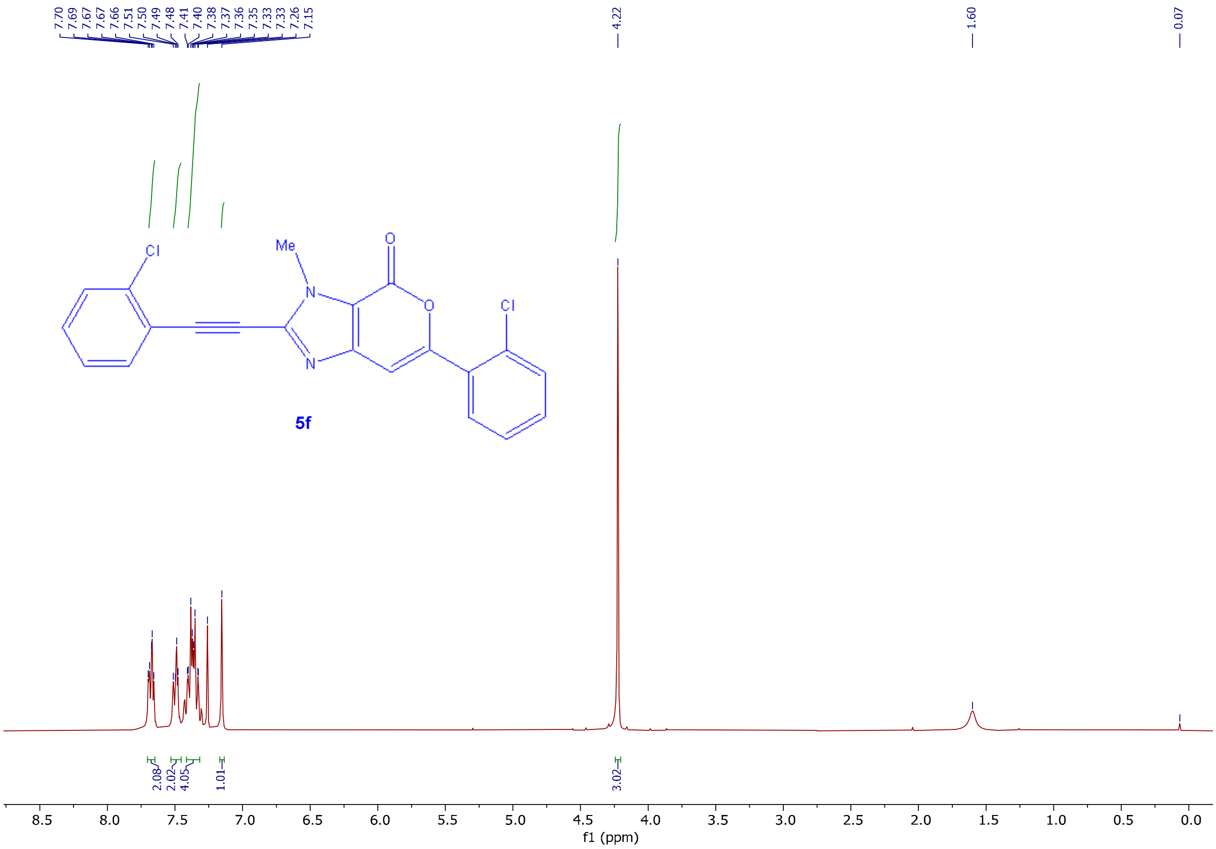

^{13}C NMR (75 MHz, $CDCl_3$) of **5f**

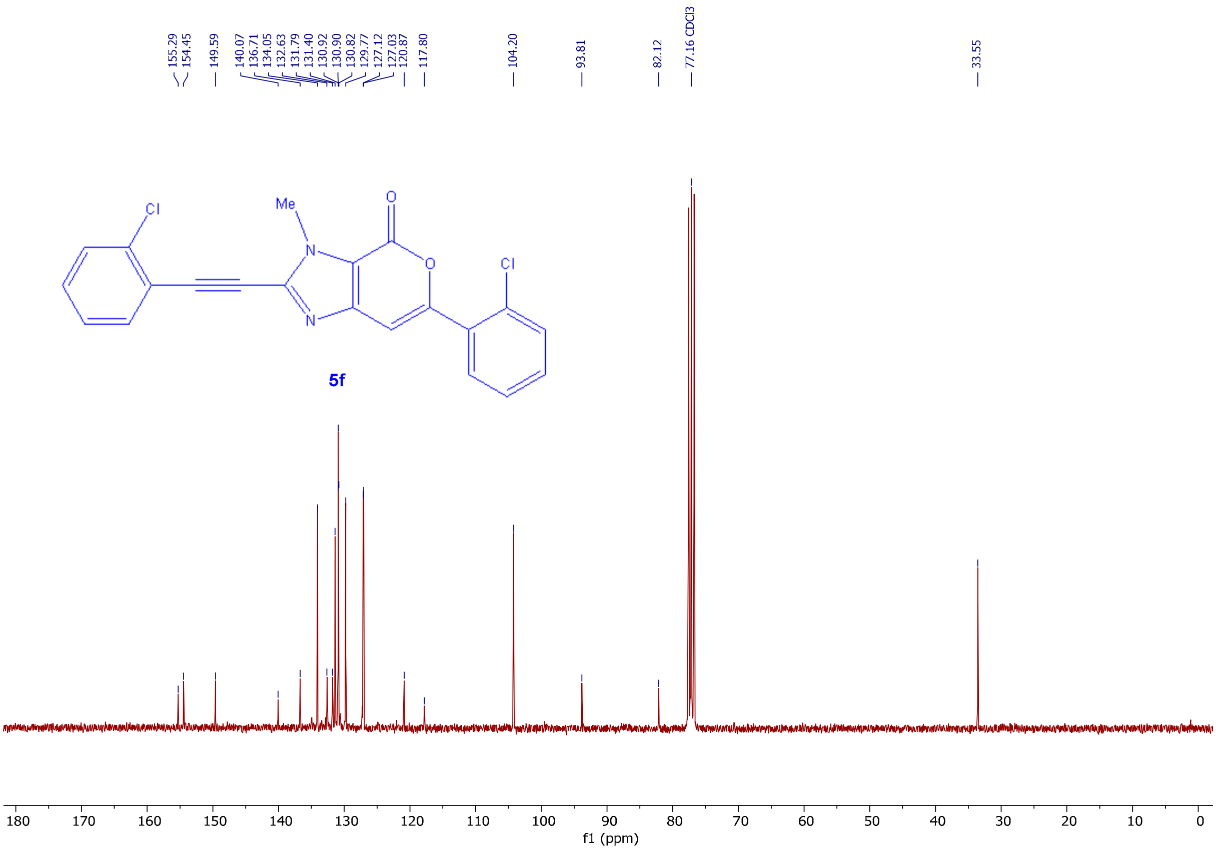

^{1}H NMR (300 MHz, $CDCl_3$) of **5g**

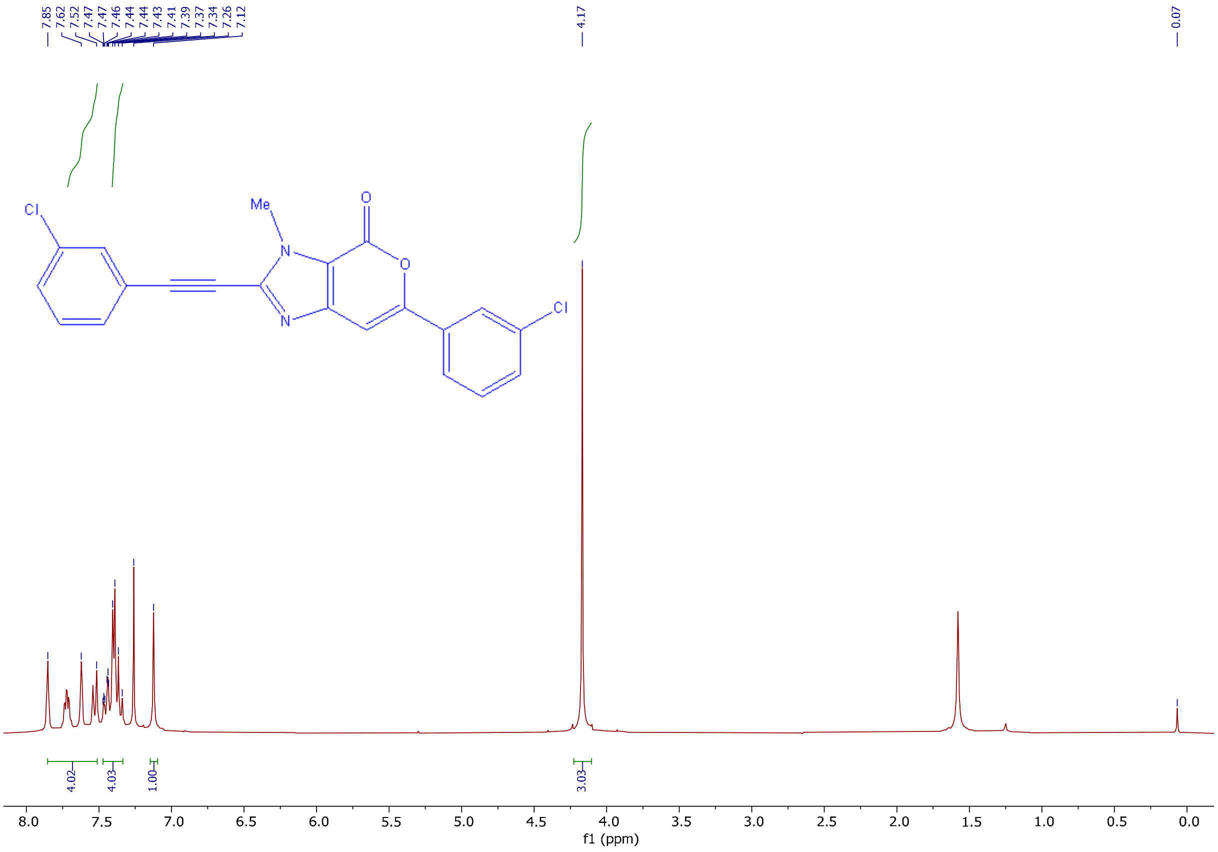

^{13}C NMR (75 MHz, $CDCl_3$) of **5g**

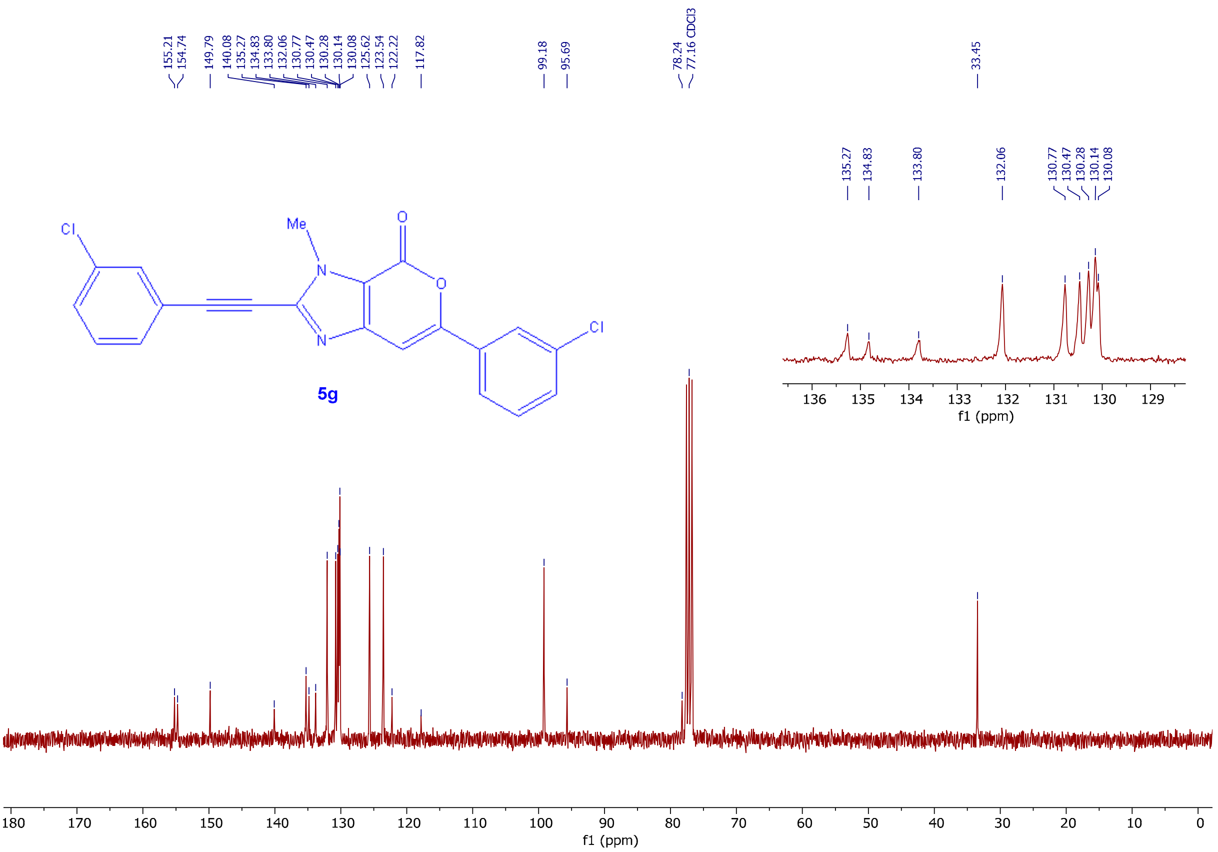

^{1}H NMR (300 MHz, $CDCl_3$) of **5h**

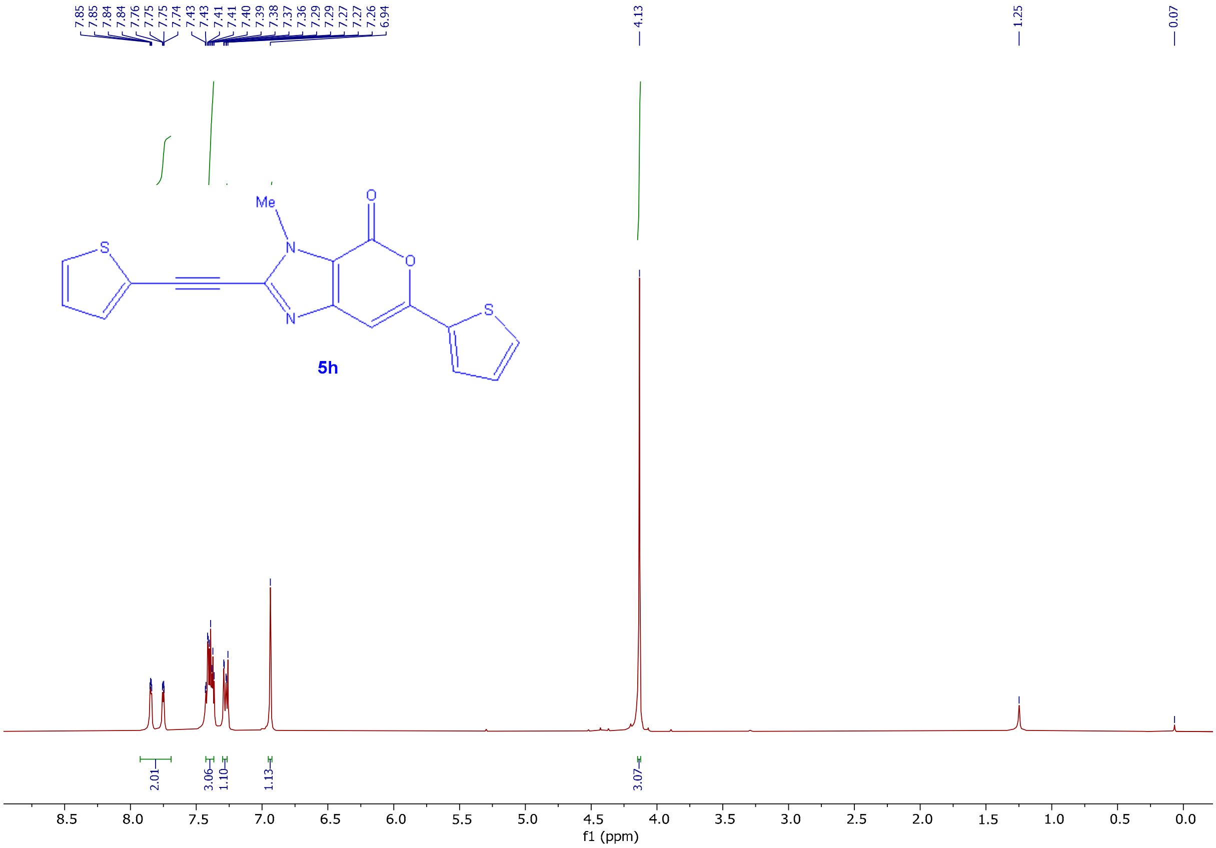

^{13}C NMR (75 MHz, $CDCl_3$) of **5h**

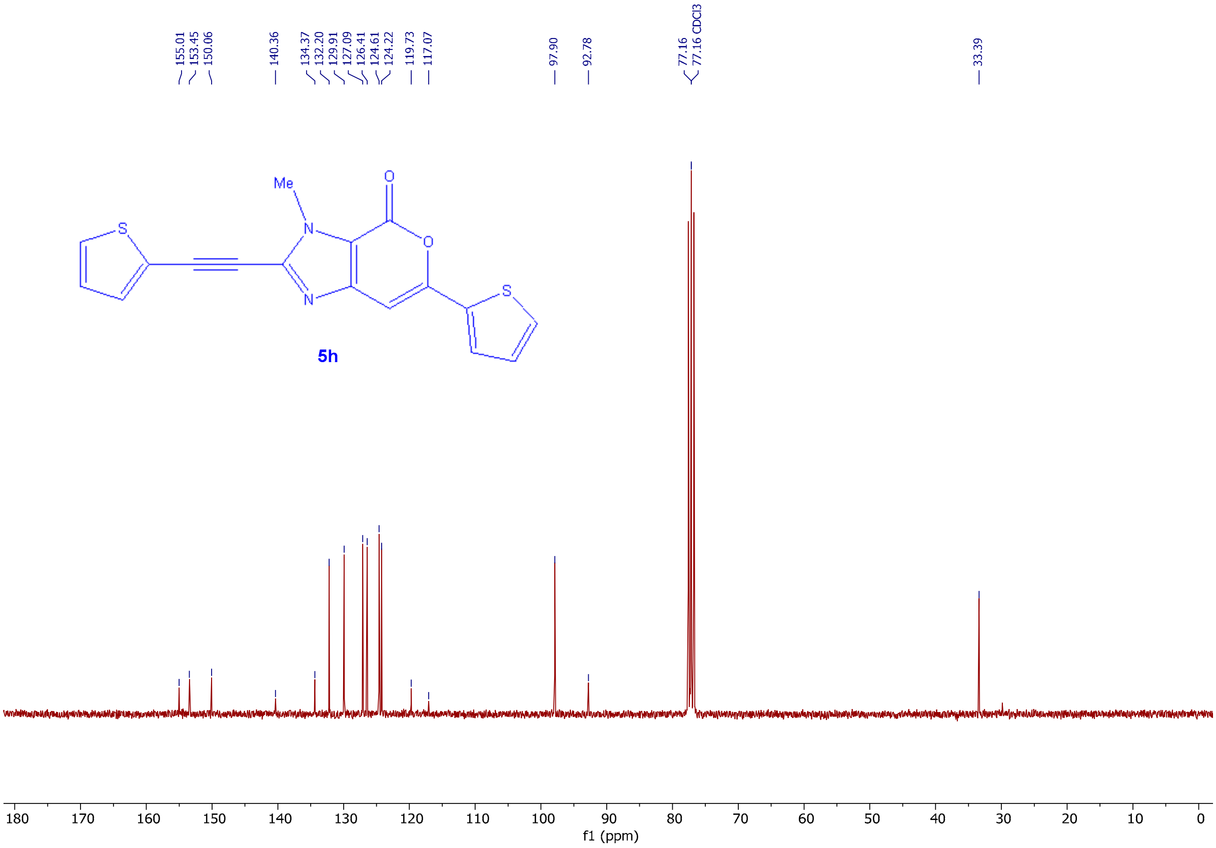

^1H NMR (300 MHz, $CDCl_3$) of **5i**

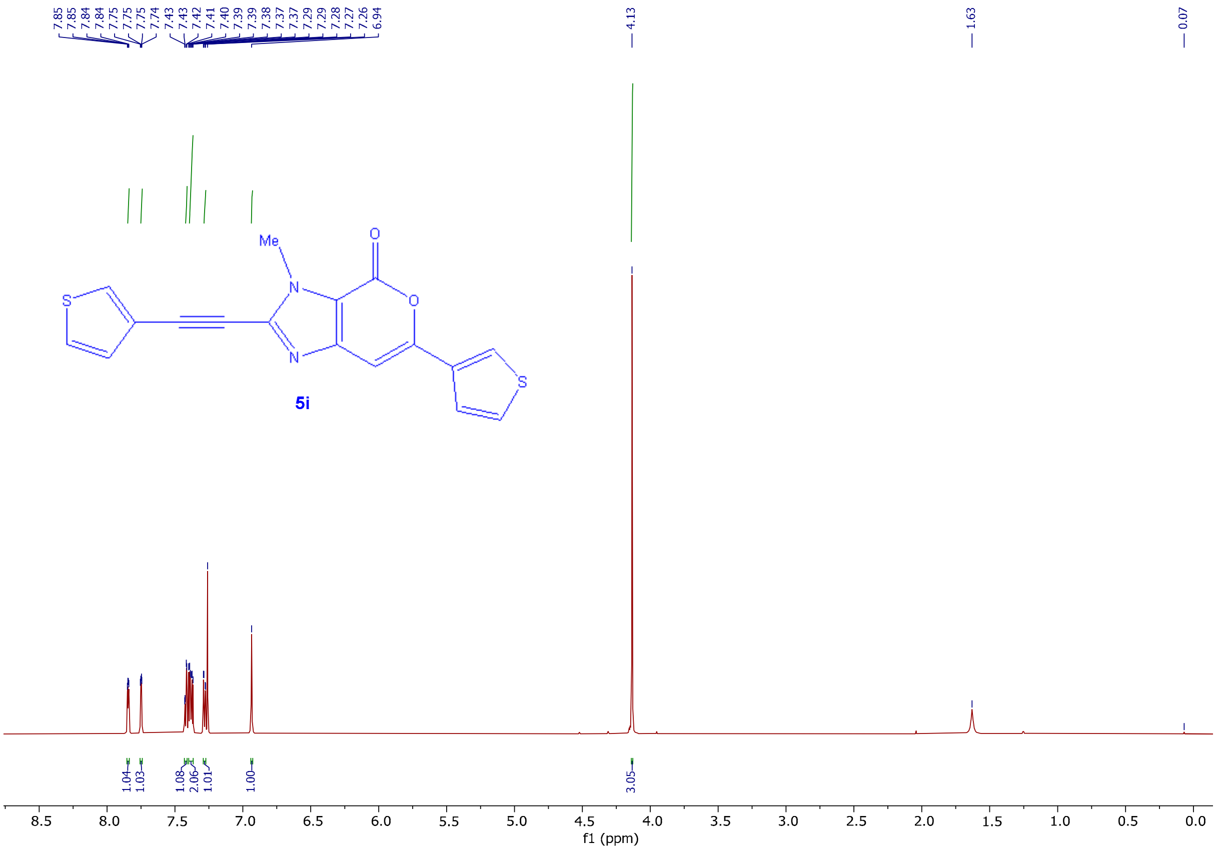

^{13}C NMR (75 MHz, $CDCl_3$) of **5i**

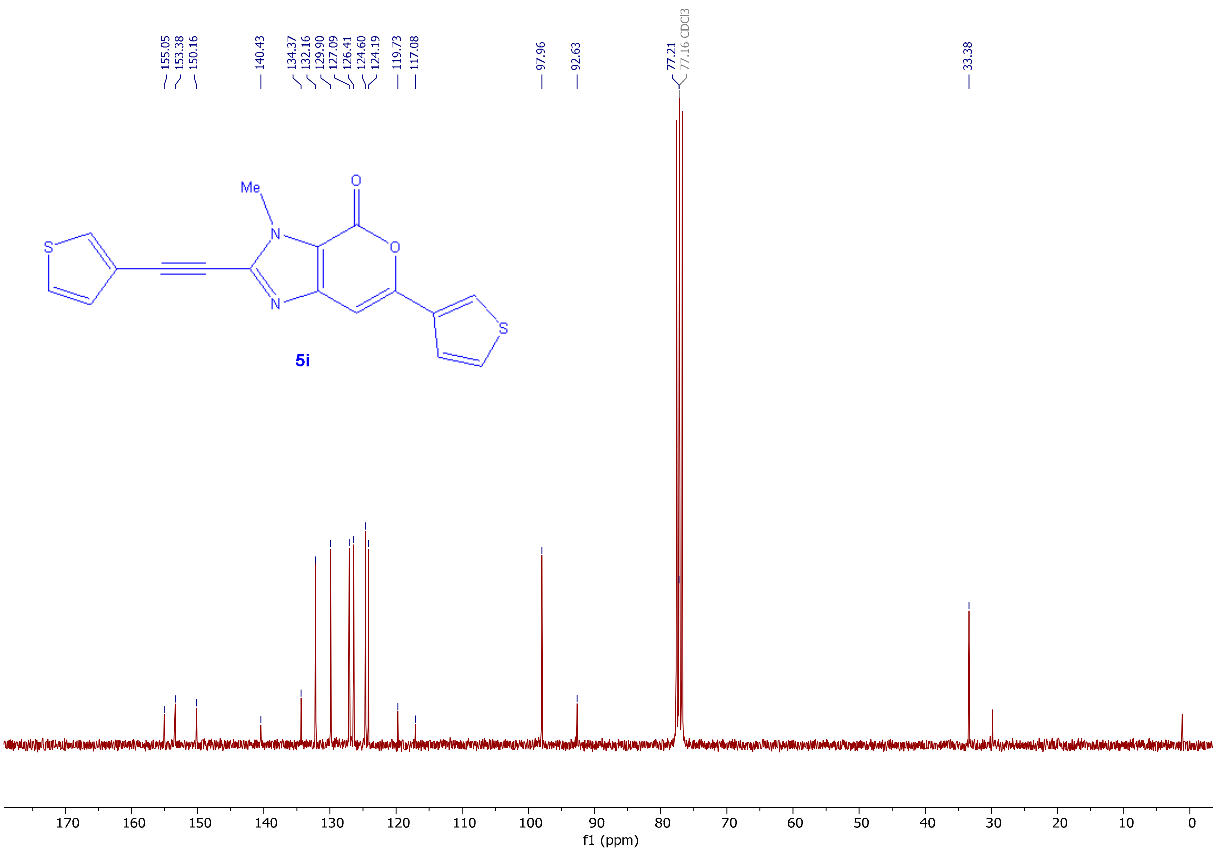

^{1}H NMR (300 MHz, $CDCl_3$) of **5j**

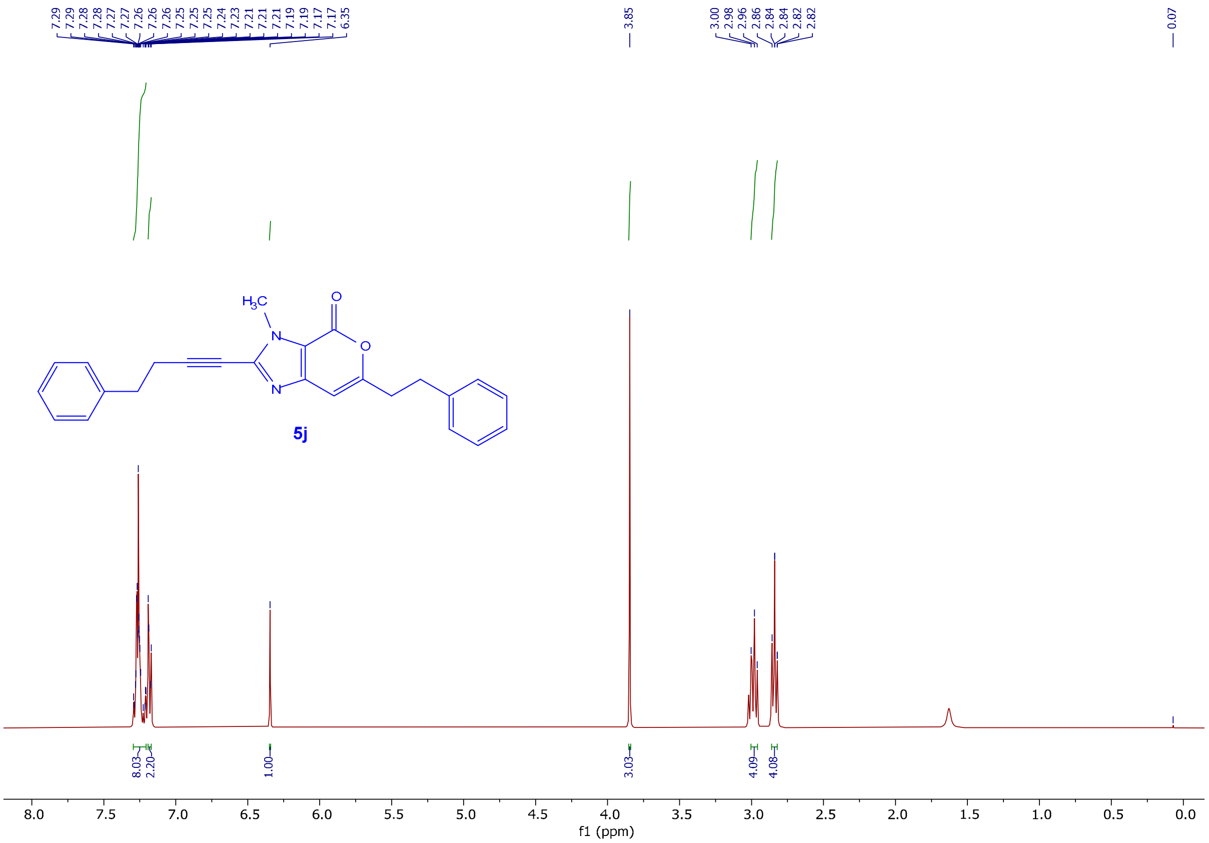

^{13}C NMR (75 MHz, $CDCl_3$) of **5j**

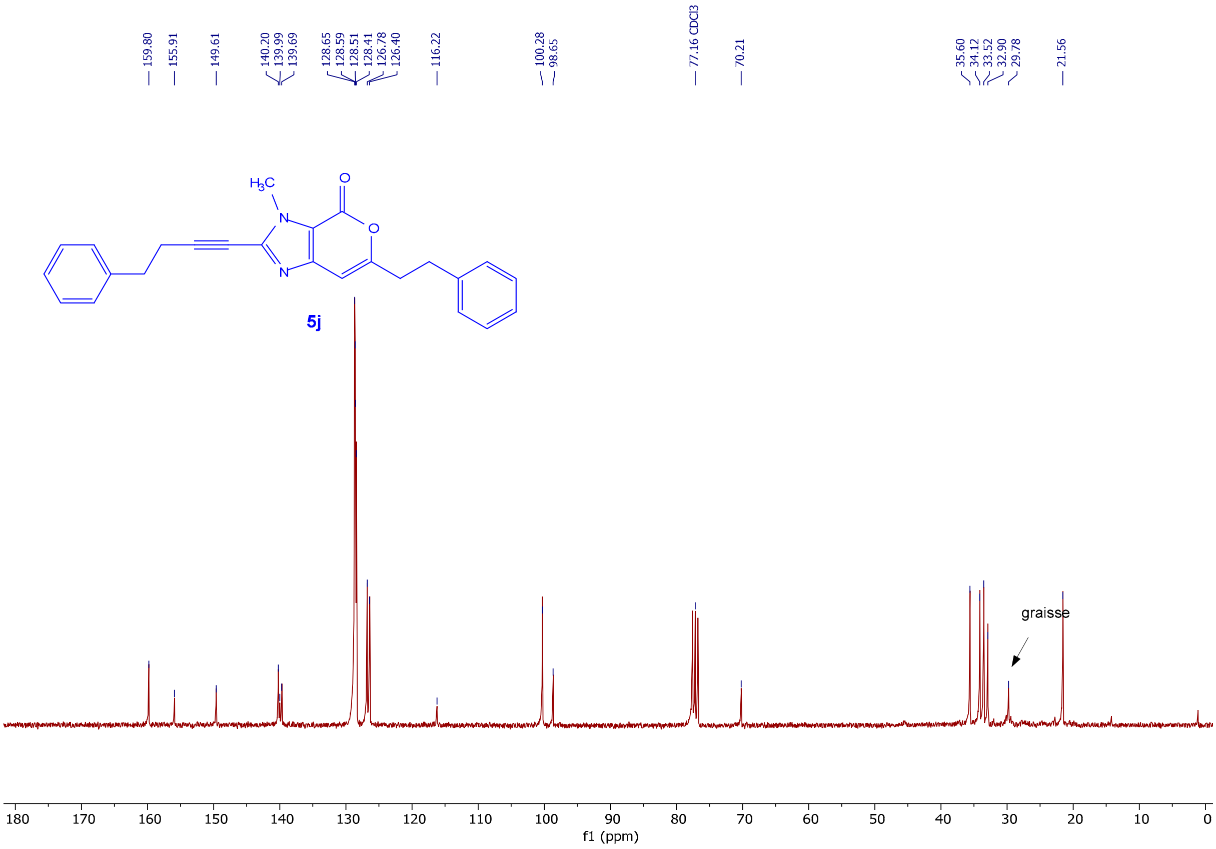

^{1}H NMR (300 MHz, $CDCl_3$) of **5k**

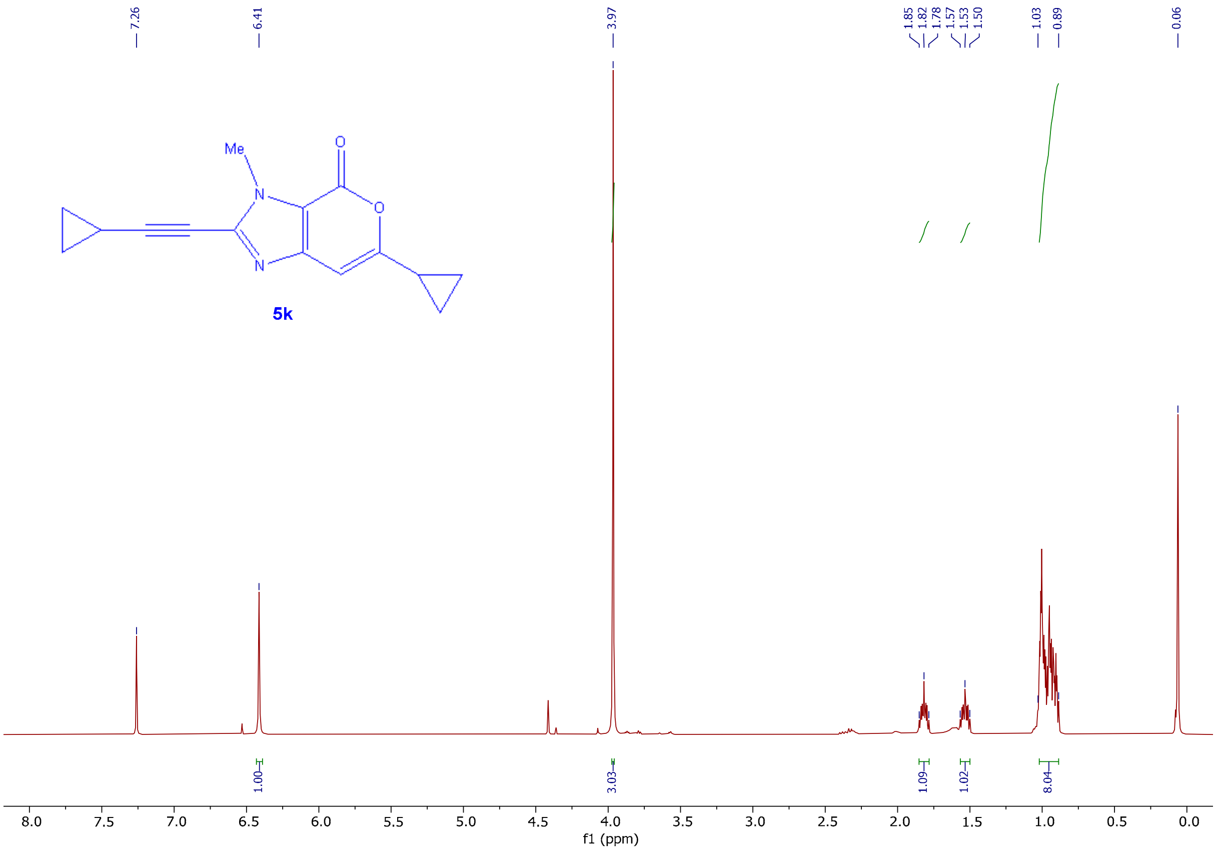

^{13}C NMR (75 MHz, $CDCl_3$) of **5k**

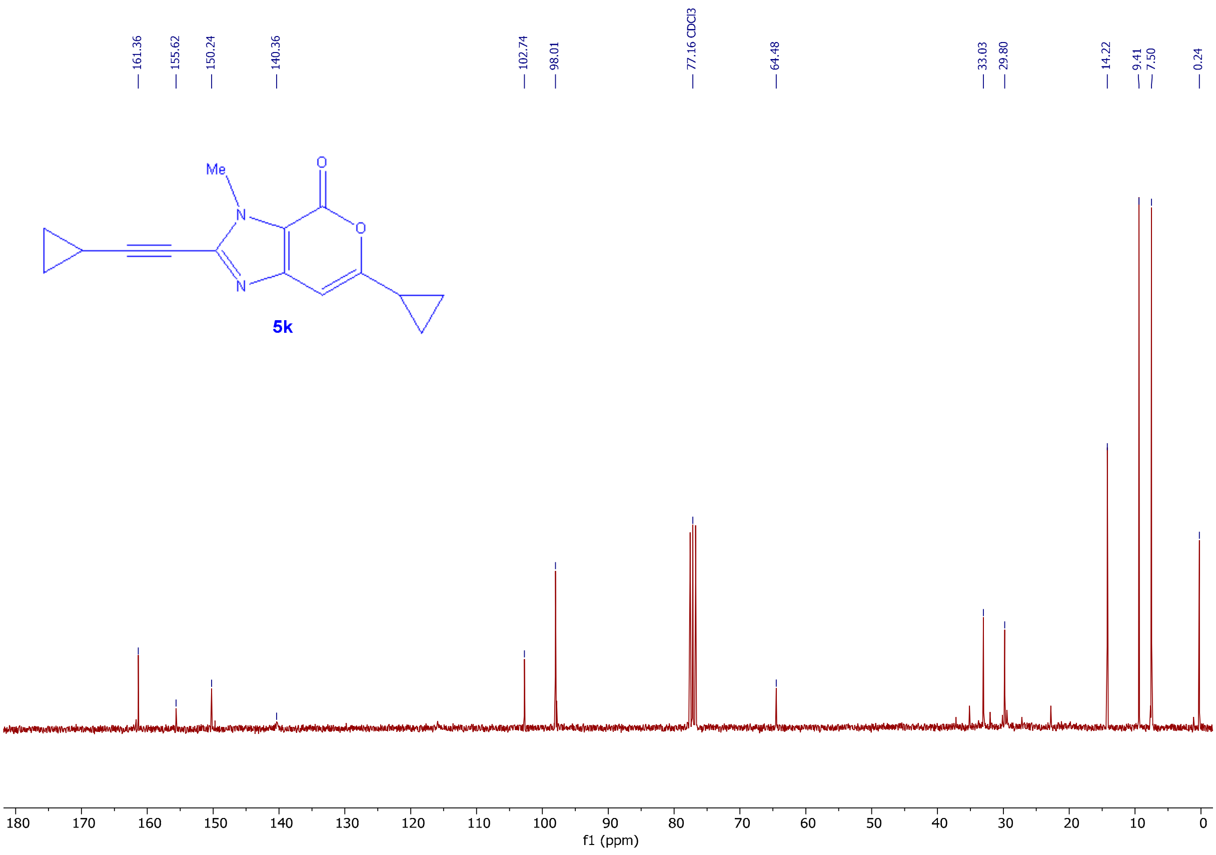

^{1}H NMR (300 MHz, $CDCl_3$) of **6**

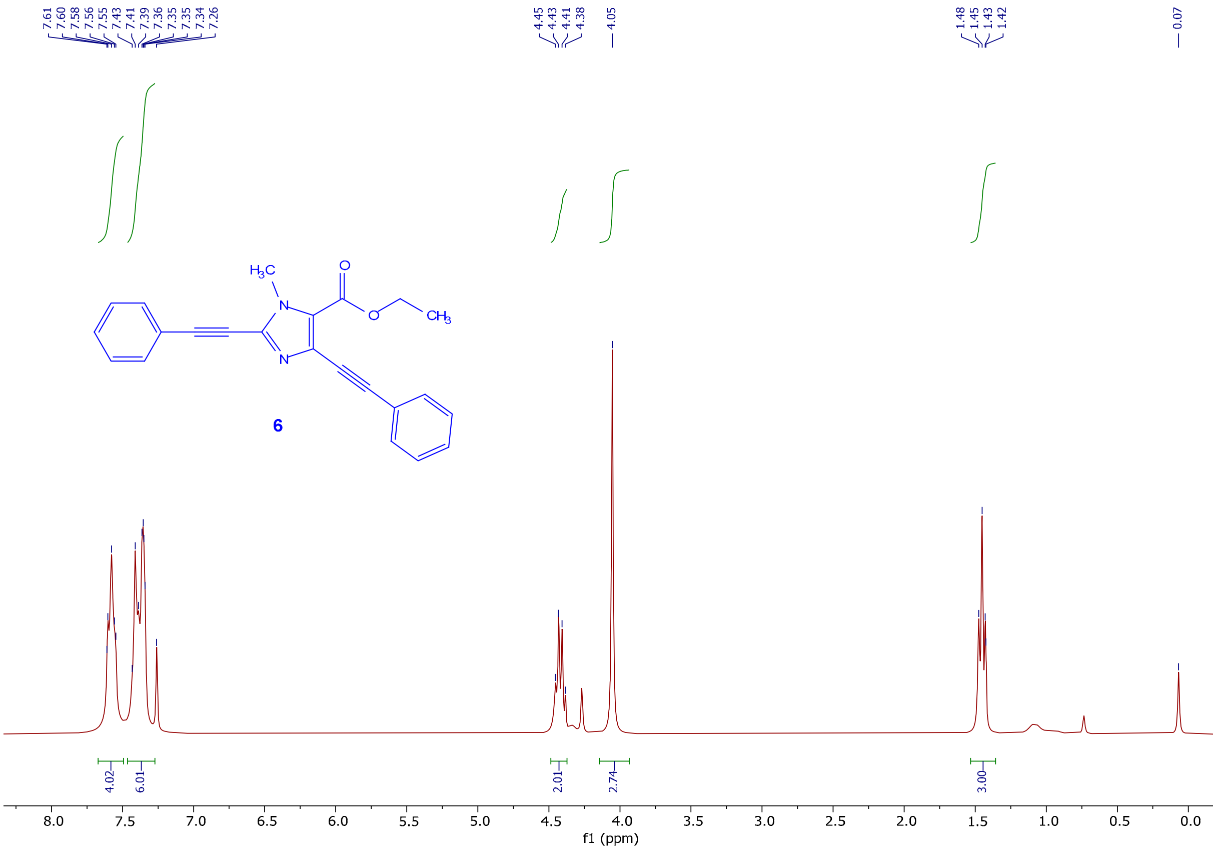

^{13}C NMR (75 MHz, $CDCl_3$) of **6**

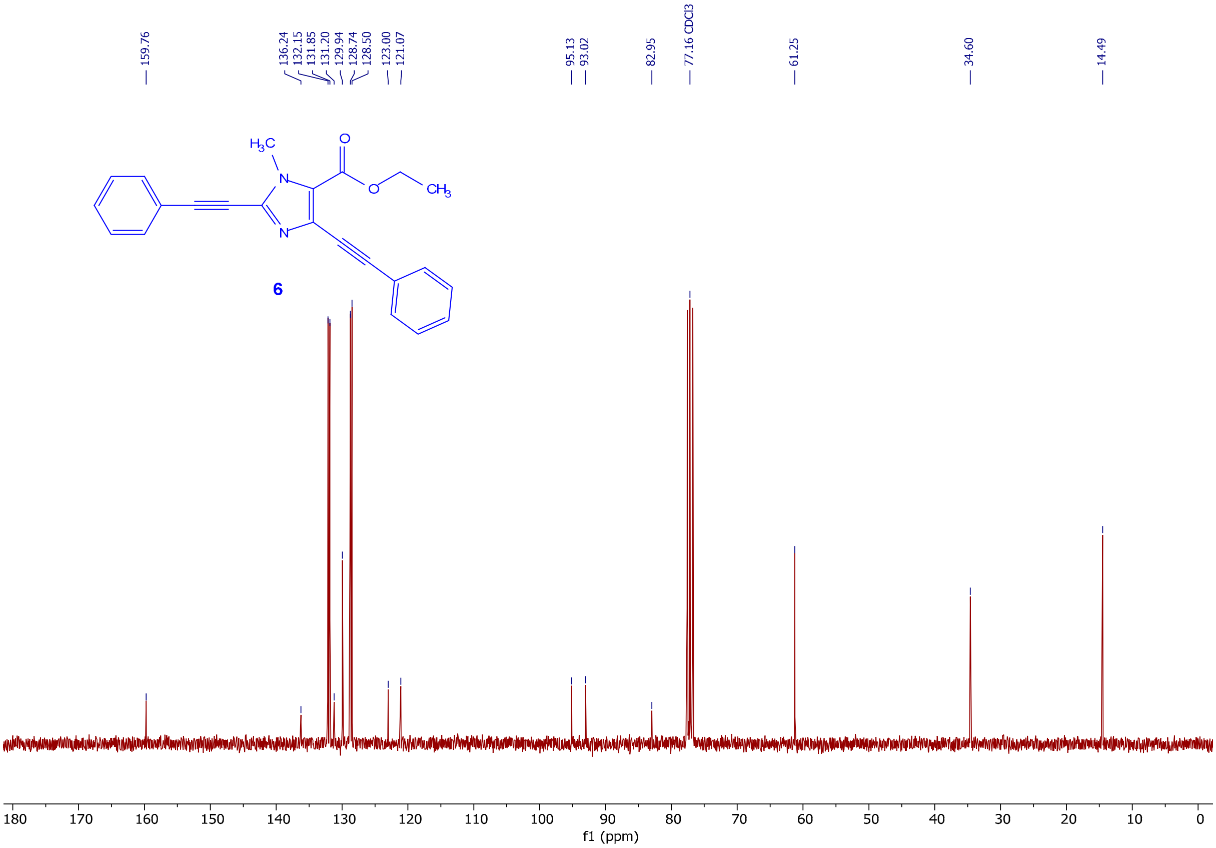

HRMS spectra of **3**

Détection mode positif :

On observe un pic unique M+1 à 406.87414 (formule brute proposée $C_7H_9I_2N_2O_2$ à -1.58728 ppm près, résolution supérieure à 100000) correspondant au composé recherché. La répartition isotopique expérimentale est cohérente avec la formule brute proposée. En haut spectre expérimental, en bas spectre théorique.

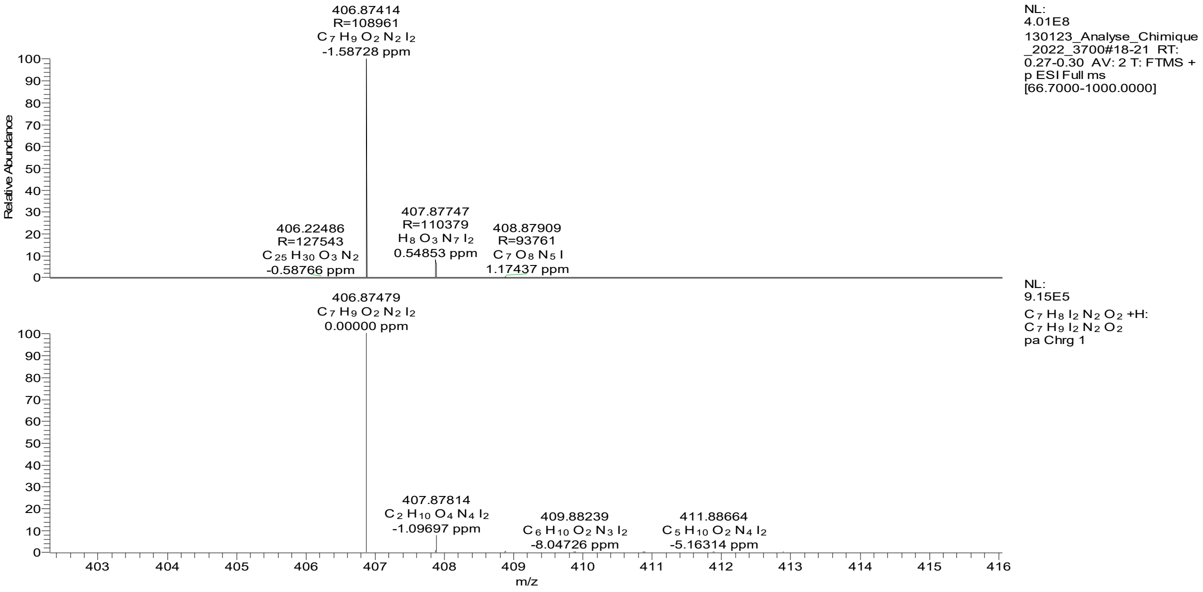

Détection mode négatif :

Aucune information supplémentaire.

Conclusion:

La masse moléculaire obtenue et le massif isotopique permettent de confirmer la structure du composé analysé.

HRMS spectra of **4**

Détection mode positif :

On observe un pic majoritaire M+1 à 378.84290 (formule brute proposée $C_5H_5I_2N_2O_2$ à -1.56057 ppm près, résolution supérieure à 110000) correspondant au composé recherché. La répartition isotopique expérimentale est cohérente avec la formule brute proposée. En haut spectre expérimental, en bas spectre théorique.

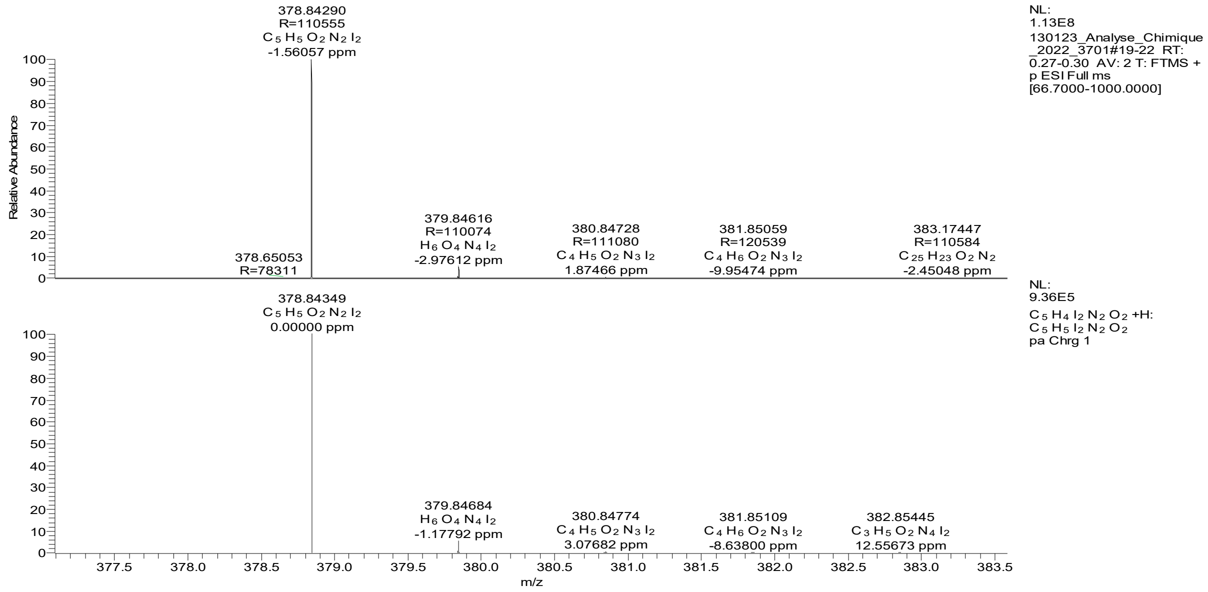

Détection mode négatif :

On observe un pic majoritaire M-1 à 376.82962 (formule brute proposée $C_5H_3I_2N_2O_2$ à 1.81462 ppm près, résolution supérieure à 100000) correspondant au composé recherché. La répartition isotopique expérimentale est cohérente avec la formule brute proposée. En haut spectre expérimental, en bas spectre théorique.

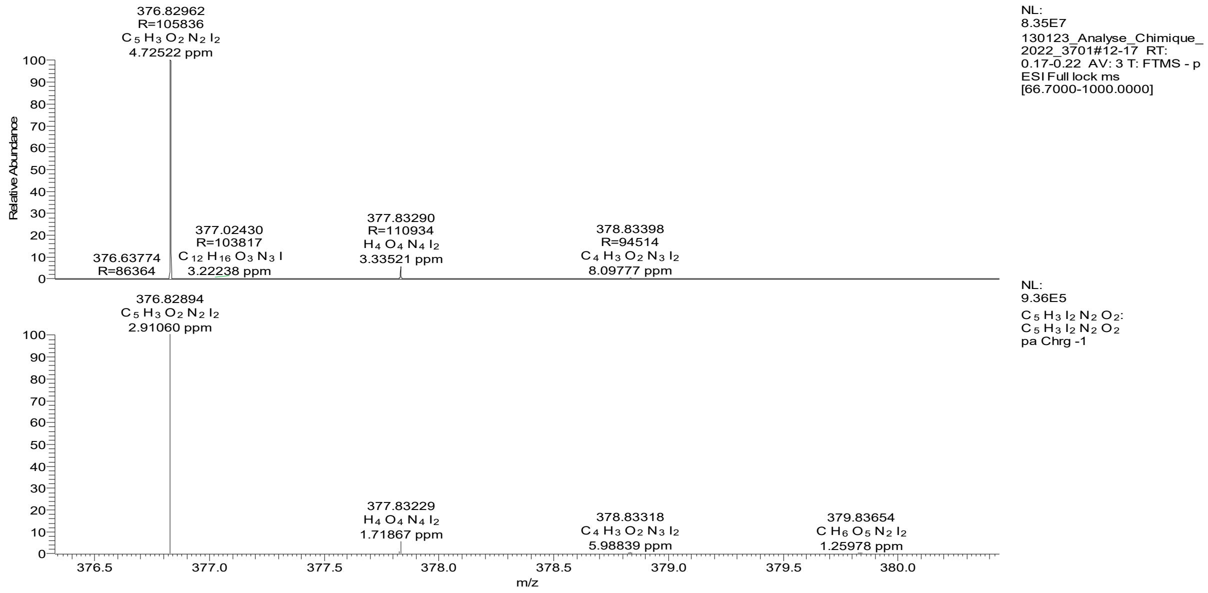

Conclusion:

La masse moléculaire obtenue et le massif isotopique permettent de confirmer la structure du composé analysé.

HRMS spectra of **5a**

Indications fournies :

Masse moléculaire : 326.3480 g.mol^{-1}

Formule brute : $C_{21}H_{14}N_2O_2$

Détection mode positif :

On observe un pic majoritaire M+1 à 327.11207 (formule brute proposée $C_{21}H_{15}N_2O_2$ à -2.25119 ppm près, résolution supérieure à 110000) correspondant au composé recherché. La répartition isotopique expérimentale est cohérente avec la formule brute proposée.
Détection mode négatif :
Aucune information supplémentaire.
Conclusion:
La masse moléculaire obtenue et le massif isotopique permettent de confirmer la structure du composé analysé.

HRMS spectra of **5b**

Indications fournies :
Masse moléculaire : 354.4012 $g.mol^{-1}$
Formule brute : $C_{23}H_{18}N_2O_2$
Détection mode positif :
On observe un pic majoritaire M+1 à 355.14334 (formule brute proposée $C_{23}H_{19}N_2O_2$ à -2.14929 ppm près, résolution supérieure à 110000) correspondant au composé recherché. La répartition isotopique expérimentale est cohérente avec la formule brute proposée.
Détection mode négatif :
Aucune information supplémentaire.
Conclusion:
La masse moléculaire obtenue et le massif isotopique permettent de confirmer la structure du composé analysé.

HRMS spectra of **5c**

Détection mode positif :
On observe un pic majoritaire M+1 à 355.14341 (formule brute proposée $C_{23}H_{19}N_2O_2$ à -1.95479 ppm près, résolution supérieure à 110000) correspondant au composé recherché. La répartition isotopique expérimentale est cohérente avec la formule brute proposée.
Détection mode négatif :
Aucune information supplémentaire.
Conclusion:
La masse moléculaire obtenue et le massif isotopique permettent de confirmer la structure du composé analysé.

HRMS spectra of **5d**

Détection mode positif :
On observe un pic majoritaire M+1 à 387.13325 (formule brute proposée $C_{23}H_{19}N_2O_4$ à -1.76464 ppm près, résolution supérieure à 100000) correspondant au composé recherché. La répartition isotopique expérimentale est cohérente avec la formule brute proposée.
Détection mode négatif :
Aucune information supplémentaire.
Conclusion:
La masse moléculaire obtenue et le massif isotopique permettent de confirmer la structure du composé analysé.

HRMS spectra of **5e**

Détection mode positif :

On observe un pic majoritaire M+1 à 439.23760 (formule brute proposée $C_{29}H_{31}N_2O_2$ à -0.92922 ppm près, résolution supérieure à 100000) correspondant au composé recherché. La répartition isotopique expérimentale est cohérente avec la formule brute proposée. En haut spectre expérimental, en bas spectre théorique.

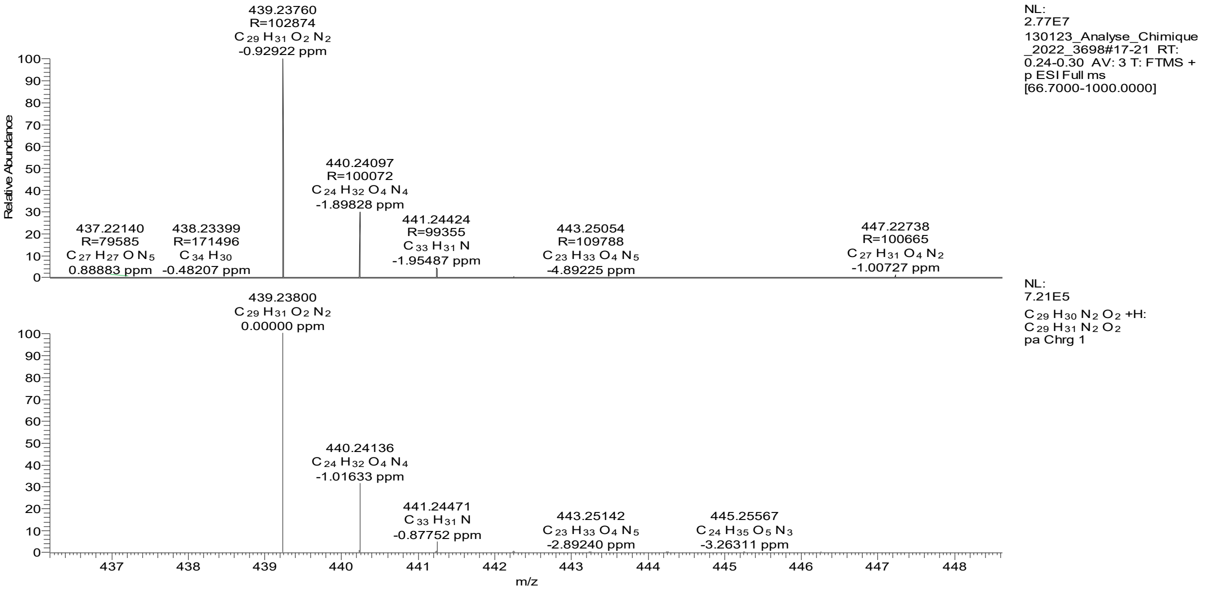

Détection mode négatif :

Aucune information supplémentaire.

Conclusion:

La masse moléculaire obtenue et le massif isotopique permettent de confirmer la structure du composé analysé.

HRMS spectra of **5g**

Détection mode positif :

On observe trois pics majoritaires M+1 à 395.03422 (formule brute proposée $C_{21}H_{13}{}^{35}Cl_2N_2O_2$ à -1.62823 ppm près, résolution supérieure à 110000), M+1 à 397.03119 (formule brute proposée $C_{21}H_{13}{}^{35}Cl^{37}ClN_2O_2$ à -1.81577 ppm près, résolution supérieure à 110000) et M+1 à 399.02820 (formule brute proposée $C_{21}H_{13}{}^{37}Cl_2N_2O_2$ à -1.89871 ppm près, résolution supérieure à 110000), correspondants tous trois au composé recherché. La répartition isotopique expérimentale est cohérente avec la formule brute proposée.

Détection mode négatif :

Aucune information supplémentaire.

Conclusion:

La masse moléculaire obtenue et le massif isotopique permettent de confirmer la structure du composé analysé.

HRMS spectra of **5h**

Détection mode positif :

On observe un pic majoritaire M+1 à 339.02496 (formule brute proposée $C_{17}H_{11}N_2O_2S_2$ à -2.012392 ppm près, résolution supérieure à 110000) correspondant au composé recherché. La répartition isotopique expérimentale est cohérente avec la formule brute proposée.

Détection mode négatif :
Aucune information supplémentaire.
Conclusion:

La masse moléculaire obtenue et le massif isotopique permettent de confirmer la structure du composé analysé.

HRMS spectra of **5i**

Détection mode positif :
On observe un pic majoritaire M+1 à 339.02526 (formule brute proposée $C_{17}H_{11}N_2O_2S_2$ à -1.12284 ppm près, résolution supérieure à 110000) correspondant au composé recherché. La répartition isotopique expérimentale est cohérente avec la formule brute proposée. En haut spectre expérimental, en bas spectre théorique.

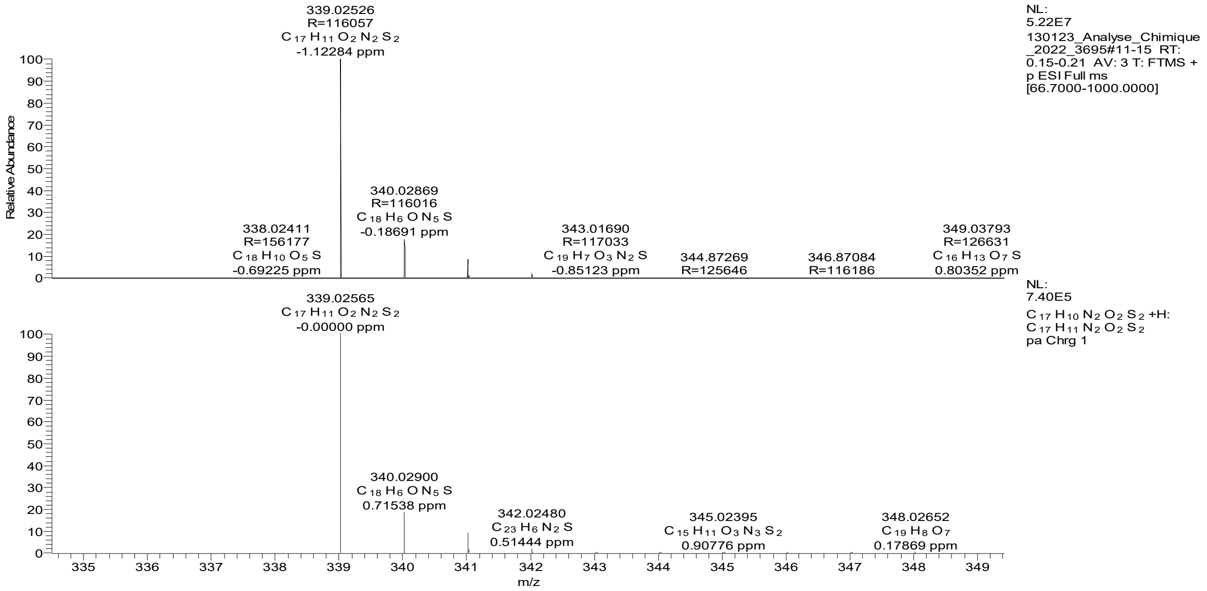

Détection mode négatif :
Aucune information supplémentaire.
Conclusion:
La masse moléculaire obtenue et le massif isotopique permettent de confirmer la structure du composé analysé.

HRMS spectra of **5j**

Détection mode positif :
On observe un pic unique M+1 à 383.17491 (formule brute proposée $C_{25}H_{23}N_2O_2$ à -1.28513 ppm près, résolution supérieure à 110000) correspondant au composé recherché. La répartition isotopique expérimentale est cohérente avec la formule brute proposée. En haut spectre expérimental, en bas spectre théorique.

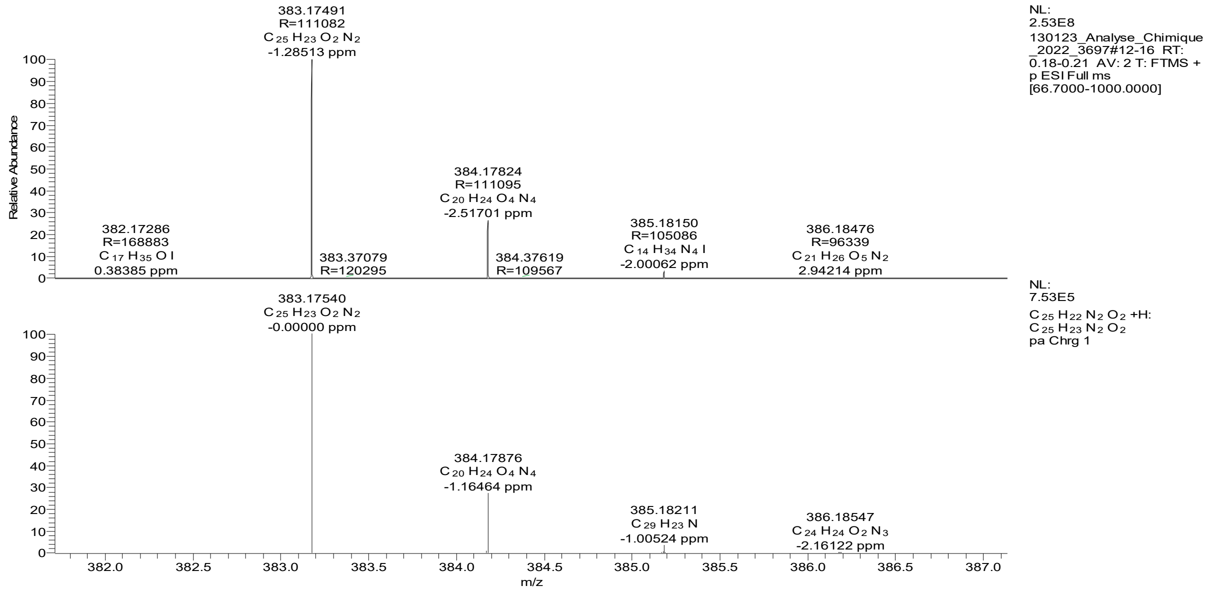

Détection mode négatif :

Aucune information supplémentaire.

Conclusion:

La masse moléculaire obtenue et le massif isotopique permettent de confirmer la structure du composé analysé.

HRMS spectra of **5k**

Détection mode positif :

On observe un pic unique M+1 à 255.11238 (formule brute proposée $C_{15}H_{15}N_2O_2$ à -1.66783 ppm près, résolution supérieure à 130000) correspondant au composé recherché. La répartition isotopique expérimentale est cohérente avec la formule brute proposée. En haut spectre expérimental, en bas spectre théorique.

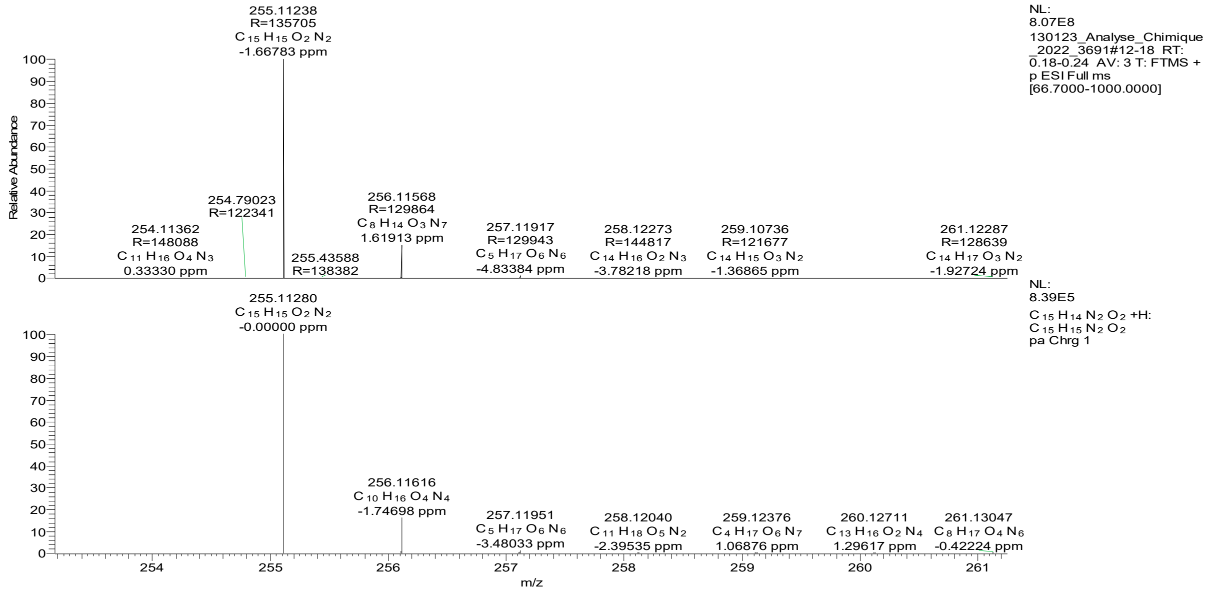

Détection mode négatif :

Aucune information supplémentaire.

Conclusion:

La masse moléculaire obtenue et le massif isotopique permettent de confirmer la structure du composé analysé.

HRMS Summary table

Ref	Formule	M + 1 Calc.	M+ 1 Exp.	δ ppm	M – 1 Calc.	M – 1 Exp.	δ ppm	Validation
**3**	$C_7H_8I_2N_2O_2$	406.87479	406.87414	-1.58728	404.86024	-	-	**Oui**
**4**	$C_5H_4I_2N_2O_2$	378.84349	378.84290	-1.56057	376.82894	376.8296<br>2	1.81462	**Oui**
**5a**	$C_{21}H_{14}N_2O_2$	327.11280	327.11207	-2.25119	325.09825	-	-	**Oui**
**5b**	$C_{23}H_{18}N_2O_2$	355.14410	355.14334	-2.14929	353.12955	-	-	**Oui**
**5c**	$C_{23}H_{18}N_2O_2$	355.14410	355.14341	-1.95479	353.12955	-	-	**Oui**
**5d**	$C_{23}H_{18}N_2O_4$	387.13393	387.13325	-1.76464	385.11938	-	-	**Oui**
**5e**	$C_{29}H_{30}N_2O_2$	439.23800	439.23760	-0.92922	437.22345	-	-	**Oui**
**5f**	$C_{21}H_{12}{}^{35}Cl_2N_2O_2$<br>$C_{21}H_{12}{}^{35}Cl^{37}ClN_2O_2$<br>$C_{21}H_{12}{}^{37}Cl_2N_2O_2$	395.03486<br>397.03191<br>399.02896	395.03422<br>397.03119<br>399.02820	-1.63823<br>-1.81577<br>-1.89871	393.02031<br>395.01736<br>397.01441	-<br>-<br>-	-<br>-<br>-	**Oui**
**5g**	$C_{21}H_{12}{}^{35}Cl_2N_2O_2$<br>$C_{21}H_{12}{}^{35}Cl^{37}ClN_2O_2$<br>$C_{21}H_{12}{}^{37}Cl_2N_2O_2$	395.03486<br>397.03191<br>399.02896	395.03429<br>397.03129<br>399.02830	-1.43889<br>-1.56370<br>-1.66191	393.02031<br>395.01736<br>397.01441	-<br>-<br>-	-<br>-<br>-	**Oui**
**5h**	$C_{17}H_{11}N_2O_2S_2$	339.02565	339.02496	-2.01239	337.01109	-	-	**Oui**
**5i**	$C_{17}H_{10}N_2O_2S_2$	339.02565	339.02526	-1.12284	337.01109	-	-	**Oui**
**5j**	$C_{25}H_{22}N_2O_2$	383.17540	383.17491	-1.28513	381.16085	-	-	**Oui**
**5k**	$C_{15}H_{14}N_2O_2$	255.11280	255.11238	-1.66783	253.09825	-	-	**Oui**

3. Details of DFT calculations

The structure of each studied species was first pre-optimized via a homemade genetic algorithm using OpenBabel python library [1] for conformer search. The strategy used was based on mutation of rotatable bounds with a final xTB optimization [2]. Then the geometry was further optimized by using the ORCA package [3] using B3LYP [4-6] and the triplet-ζ basis set def2-TZVP [7], optimized in DMF solvent using SMD solvation model, with a convergence criterion of 10^{-8} Hartree, using the hybrid functional, to collect its more stable 3D conformer. Frequency calculations on these optimized geometries confirmed the absence of any imaginary wavenumber values, thereby verifying that the optimizations successfully reached true minimum geometries.

Eventually, single point calculation was performed from optimized geometry in the same solvent condition and level of theory to obtain Mulliken Atomic charges.

3.1. Cartesian coordinates of the molecule 5a

	X	Y	Z
C	1.38374406097338	0.54219517041893	0.66244156920403

N	2.79215870315966	0.31486600920037	0.36052389151248
C	3.72193680282658	1.3027411065795	0.13951059712606
C	3.40388180864584	2.67615823850169	0.12988335408766
C	3.13709059144703	3.85669212749738	0.11573130235514
C	2.82586719288196	5.2436091895185	0.09543385358014
C	1.51156683858664	5.68278801867791	0.32951066891879
C	1.21400054472585	7.03789601152565	0.30707019034846
C	2.21334882164024	7.97407972651922	0.05228269782087
C	3.5185092306867	7.5471490933109	-0.18054460099141
C	3.82829639688795	6.19472996135632	-0.16034904049907
N	4.93096636772066	0.79421338333396	-0.04797523330762
C	4.78012518460268	-0.56670013103014	0.07892519218318
C	5.87739478549105	-1.44057465067173	-0.08846821496849
C	6.84683367468533	-2.1482544639558	-0.24843954967286
C	7.97532682615859	-2.99600428198971	-0.42854004914848
C	9.24599164831842	-2.4525831121306	-0.68595204226904
C	10.3431242279942	-3.28482560442187	-0.85954019495117
C	10.1980771014554	-4.66785776240262	-0.78137419798108
C	8.94305044637349	-5.21623620361995	-0.52745291131325
C	7.83947539668689	-4.3931447989935	-0.35178003460233
C	3.44554085542918	-0.88619754799081	0.33592104177783
C	2.77658347430062	-2.24703244902703	0.49865239909978
O	1.67348298540298	-2.38680303585724	-0.07792292757879
O	3.41411958145183	-3.07799668302441	1.17620340047007
H	1.01249078408762	1.36153372207399	0.04970547811299
H	1.26133085956068	0.79901012097106	1.7161799639196
H	0.83928508200247	-0.36872510869441	0.43420119715201
H	4.84293787843963	5.863566009394	-0.34131710473216
H	0.73413059565549	4.95585167238772	0.52697667854039

H	0.19738240855016	7.36468671851678	0.48858661011294
H	1.97623803544907	9.03069651364346	0.03533969517206
H	4.29933213008902	8.27111060309183	-0.37906979101497
H	8.82265077255733	-6.29107776576536	-0.46566776562053
H	9.36202826404224	-1.37786312328319	-0.7473130162643
H	6.86506678747726	-4.82209045794511	-0.15467224968603
H	11.0567329937209	-5.31373285759372	-0.91762660132548
H	11.3161638598347	-2.85132935812207	-1.05687625556744

3.2. Cartesian coordinates of the molecule 5b

	X	Y	Z
C	1.51538018445146	0.38192190701372	0.0577024837348
C	2.99982287998285	0.23583147356669	-0.13409483692866
C	3.80115657899517	1.33287887823884	-0.46075189266808
C	5.16761903244159	1.19652082614556	-0.66182346238554
C	5.78550063546405	-0.0581260124558	-0.53938355411855
C	7.18582206896028	-0.20845494594779	-0.74060251654506
C	8.37616930162069	-0.35666724591251	-0.9071400300986
C	9.77005197876309	-0.46880064927553	-1.1107108306572
N	10.535769651154	0.63855456978377	-1.39201088450657
C	11.7709874427724	0.18405956522741	-1.54704165147165
C	12.9044878915933	0.97924010793256	-1.81423018277304
C	13.8711636437155	1.67086417616217	-2.0446363826689
C	15.0008790406513	2.4893339082565	-2.31420754161079
C	16.2733424073906	1.92365672407189	-2.49302663336763
C	17.3695176743954	2.73133169208785	-2.75695975072355
C	17.2447351488497	4.12048204043215	-2.85144643087368
C	18.4412579474378	4.99366042812258	-3.10881793828832
C	15.973481030969	4.67766733889099	-2.67573322607694

C	14.868150231013	3.88489766368026	-2.4112814487391
N	11.8244898073413	-1.18058084544429	-1.39956649007537
C	13.0440097985441	-1.97881375706692	-1.44805504742974
C	10.5601079501631	-1.61950200249993	-1.11087542749657
C	10.1652473927823	-3.07845661337237	-0.90860497070178
O	10.7221367480412	-3.90865727248799	-1.66389291275156
O	9.3130827643183	-3.28265133069984	-0.02041215214369
C	4.98341585512341	-1.16418179126065	-0.2082131357187
C	3.62012942375687	-1.01227284214064	-0.01069615913699
H	1.23651812792885	1.42406338751196	0.22012769220622
H	1.16472667038616	-0.20517200915241	0.90944393967255
H	0.96986681285946	0.02707528503675	-0.82264142169376
H	3.02389861633247	-1.88066803798818	0.24706969327141
H	3.34838480325812	2.31324649940639	-0.55777825807609
H	13.8935730584479	4.33817489031734	-2.2803635936115
H	16.3943391189196	0.84979049747852	-2.42656926522809
H	19.2475815743212	4.43350734034559	-3.58458088706462
H	18.8335210971137	5.40248170251253	-2.1718349531156
H	18.1848749215785	5.84176744319392	-3.7468811720001
H	5.44017559544754	-2.14066580029152	-0.1055934304133
H	15.8474273300773	5.7520151477497	-2.74936529484196
H	13.6716955543964	-1.62937534286003	-2.26604782821997
H	12.7610857361896	-3.01337755834629	-1.61445254040623
H	13.5933384542689	-1.88485457690082	-0.50963285972562
H	18.3422363162636	2.27304706843444	-2.89492908561264
H	5.76656070151894	2.06310707250342	-0.91288572891925

3.3. Cartesian coordinates of the molecule 5c

	X	Y	Z

C	1.1931931491393	-0.25560470900001	0.29171078512644
C	2.66996022377458	-0.37168714675949	0.0223706979767
C	3.4796334830762	0.76981690255895	-0.01082993404397
C	4.84374664979397	0.66679935369069	-0.25840413977344
C	5.43081800013102	-0.57247142721769	-0.47757748931291
C	4.64082908637548	-1.73184530348123	-0.44936872787154
C	5.22104261054216	-3.01251465794108	-0.67038885186006
C	5.69852527060259	-4.11014037016445	-0.85398879473202
C	6.30697065782964	-5.36433762435309	-1.08386749102313
N	7.65616788547334	-5.46479937457627	-1.33070015405328
C	7.88489834370871	-6.75553088542746	-1.52414152181179
C	9.14670930857781	-7.33215745726052	-1.77553001710809
C	10.233704379099	-7.82061518254385	-1.98826939047386
C	11.5122950987715	-8.39026421228721	-2.23760016194085
C	11.6590833044062	-9.77848953931537	-2.37658038198514
C	12.9170440973116	-10.3137104959069	-2.61830282709015
C	14.0289762989865	-9.48603756919736	-2.72417913169629
C	13.9068673301206	-8.09792165506432	-2.58989500284273
C	15.1151861399104	-7.2077716792494	-2.70672867130983
C	12.6451423768452	-7.56426532078952	-2.34704579441611
N	6.72580713968188	-7.48833838078655	-1.43414877024741
C	6.64385956783139	-8.94065091048524	-1.53782692518853
C	5.70668442385201	-6.62325795862641	-1.14373236919082
C	4.24150948740892	-7.01837149088265	-0.99299811522844
O	3.81616124823553	-7.86893242154787	-1.80838411025476
O	3.62294087448776	-6.4346280069247	-0.08027643692541
C	3.26201527959843	-1.61165921750521	-0.19761591227773
H	0.70537801140864	0.37792963255352	-0.45402279792956
H	1.00581611929955	0.19861813862149	1.26855029752159

H	0.70826187223904	-1.23228799567159	0.27495730397373
H	6.49314040476968	-0.6510921998777	-0.67022820691394
H	3.03599204914931	1.74471628931916	0.15812474676791
H	2.65296790790687	-2.50743003596548	-0.17624757434332
H	15.004645602972	-9.91964621171761	-2.91326683944128
H	12.5266746797433	-6.49283478949826	-2.23954647983428
H	13.0304845822793	-11.3858026659722	-2.72519629447252
H	15.8659170962831	-7.46282516219449	-1.9539129063701
H	14.8492025411219	-6.15816847238795	-2.5773802031818
H	15.592087146437	-7.31882983702965	-3.68429255476801
H	10.7930580638592	-10.4223914619399	-2.29447227025299
H	7.28732967358181	-9.28148561525983	-2.34707290515437
H	5.61242988119382	-9.20457378835164	-1.74927575192157
H	6.96692939568164	-9.40376837951921	-0.60386668440576
H	5.45646425650177	1.56017829593584	-0.28083823971854

3.4. Cartesian coordinates of the molecule 5d

	X	Y	Z
C	0.96604037995869	0.50465551267907	-0.20793198549306
O	2.31385664505358	0.12624381692442	-0.49231385530176
C	2.93135869436545	0.67880999641672	-1.56478467106055
C	4.25030336414987	0.26806879582817	-1.79730576250283
C	4.96317657980296	0.77369532570442	-2.86665932056911
C	4.38445546348531	1.71047489303337	-3.74448760143541
C	5.11631360927117	2.2329550487647	-4.84619441748942
C	5.72196263179774	2.69243145510512	-5.78917967541029
C	6.47691375353377	3.18603425352091	-6.87773002167587
N	7.74895281990412	2.72273020936101	-7.12362823485338
C	8.15317927858901	3.36057755466496	-8.21329918516409

C	9.4218086384596	3.22299071979735	-8.81396438666358
C	10.5149382624617	3.09274476074153	-9.31880346405836
C	11.7977505623801	2.93557137087294	-9.90630590809879
C	12.726642939931	2.03065891740852	-9.37342224707491
C	13.982919006021	1.86931105935616	-9.94149731316253
C	14.3384930176182	2.61871728415691	-11.0667475051006
O	15.5343467644986	2.53623786291799	-11.6946838379459
C	16.5127413431265	1.62245477179685	-11.1935038014412
C	13.4188854329925	3.52706185049793	-11.6086587378894
C	12.1712440178737	3.68329137400599	-11.039724577587
N	7.17860579848538	4.20038317258999	-8.69204751434945
C	7.33219812053019	5.09056782062795	-9.83661541476379
C	6.10096609912387	4.11418691809865	-7.84893762496312
C	4.78303173177227	4.85691504065618	-8.03342516626932
O	4.37953159063261	4.98112928758011	-9.21353253171957
O	4.23692978583152	5.24487767657728	-6.98036452769416
C	3.0640880819429	2.11325343399445	-3.49876536526797
C	2.34118373566399	1.60819147094697	-2.42542055076489
H	0.6803933343239	-0.05657629008235	0.67947382431551
H	0.29749317660802	0.24236423335477	-1.03191977407798
H	0.88977528706596	1.57485840115538	0.00079803354469
H	4.70215202955707	-0.45216116620543	-1.12645591390303
H	5.98172810812819	0.44613148829904	-3.03292925543833
H	12.4607077934709	1.44525309835165	-8.50227551598928
H	14.6719740397326	1.16256816805944	-9.50217740419205
H	17.376293248646	1.72412084052887	-11.847485798565
H	16.1491350995739	0.59255448494682	-11.2316077668368
H	16.8023548786862	1.87354940042467	-10.1700732901508
H	13.7021874954916	4.1038096391268	-12.4803587402753

H	11.4709453023715	4.38829911127633	-11.4692510995402
H	7.85373588807479	4.56505393003644	-10.6348395269733
H	7.90839509820829	5.97339571366453	-9.55341977306348
H	6.34117420409029	5.38188115994988	-10.1699893023955
H	2.59610308357896	2.8328175660128	-4.15880601048355
H	1.3258257831287	1.94386756646975	-2.2703424801984

3.5. Cartesian coordinates of the molecule 5e

	X	Y	Z
C	1.5830982695344	0.56886449577742	1.00223815842023
N	3.02213998745355	0.37675949654305	0.86529942669833
C	3.93174228296906	1.3796248667512	0.63269576635744
C	3.57697397150246	2.74177770570143	0.54803140644125
C	3.27978315401722	3.91327010646487	0.47597295626053
C	2.93597881779427	5.28940529416926	0.39444114148199
C	3.91273934212798	6.26295908611298	0.14568923290039
C	3.56964620789746	7.60689295857206	0.06885549093469
C	2.25029401172253	8.03969396593298	0.2341670282101
C	1.83922914837708	9.51639354987617	0.15584360484949
C	3.03178233378912	10.4418136899899	-0.12374866206371
C	0.80723941974986	9.70119329265464	-0.9764487449221
C	1.20078217944124	9.94072417019076	1.49506290968901
C	1.2815961201537	7.05532473477071	0.48245443060325
C	1.60636071020411	5.71225910950152	0.56223874998643
N	5.15730361986812	0.8962687188054	0.48905663019679
C	5.03007098729014	-0.46796989380107	0.61005907777012
C	6.1593155439871	-1.31390265616588	0.5325922422111
C	7.15725594536324	-1.99639031159063	0.46133967398979
C	8.31412899162249	-2.82016703961528	0.37974976649776

C	8.20969865286006	-4.22030420853631	0.44895077087632
C	9.33924051777875	-5.01722098196922	0.37009209578686
C	10.623360717014	-4.47217424588172	0.21972339278674
C	11.8458169024814	-5.39722570814221	0.13557298894401
C	11.9298187338513	-6.25621633838401	1.41466810415142
C	11.7003652932341	-6.32704820626801	-1.08732488483854
C	13.1608552796847	-4.61754577724915	-0.00646252458349
C	10.7165351876741	-3.0788472918737	0.15138262478444
C	9.59225049197334	-2.26551383639415	0.22895341334831
C	3.69853935491645	-0.81337071995686	0.8464536335981
C	3.07190355497043	-2.17955892112473	1.10393083942276
O	2.19639249845237	-2.22251542152847	1.99918685279138
O	3.51582304831017	-3.1115237159733	0.403278900067
H	1.3927995533263	1.44804341859244	1.61527212622227
H	1.1712435677728	-0.31337834362579	1.48194304448429
H	1.12721049823771	0.71131031002013	0.02081876073486
H	0.24604407377369	7.34069031657991	0.61775381327437
H	4.94461081238691	5.96267177379797	0.01281091547493
H	3.51209357930236	10.2132191822613	-1.07792146425067
H	3.78873659099589	10.3818110214405	0.66157595464039
H	2.68463277285272	11.4764402614025	-0.16889104770272
H	1.22798856306783	9.41234194767878	-1.94293996151398
H	0.50519949779626	10.7498389162384	-1.04016733546735
H	-0.09156384037842	9.10529622845471	-0.80796738843519
H	1.90795611858561	9.82833425633987	2.32079398702013
H	0.31392243939832	9.34847675158577	1.72741019855105
H	0.89901400592805	10.9904269914058	1.45017264426828
H	0.83303010856531	4.97939878079659	0.75544947343833
H	4.35842227792179	8.32023150333039	-0.12430973512104

H	11.6793622733797	-2.60120799192131	0.0356134250716
H	7.23448897218687	-4.67657249459783	0.56582728370552
H	11.0407328731255	-6.87522425955641	1.54740953015334
H	12.0403335886492	-5.62907457256634	2.30301547134931
H	12.7948602410851	-6.92265464855368	1.36282777727551
H	10.8060325784327	-6.94914984457214	-1.0197275528159
H	12.5648941265269	-6.99240088061577	-1.15782101597346
H	11.641827246044	-5.7512752152926	-2.01453269882727
H	13.3379394691748	-3.95594462332669	0.84464058554899
H	13.1831302142501	-4.01427807076099	-0.9169196474233
H	13.9951524604527	-5.32080603788596	-0.05669592302762
H	9.70371182849928	-1.18968204901567	0.17298968367494
H	9.21261623059316	-6.0910455949935	0.42786760202225

3.6. Cartesian coordinates of the molecule 5f

	X	Y	Z
C	1.0177848625505	0.42499532791599	0.23321269619679
N	2.43858136525608	0.10034922189438	0.17526309515343
C	3.45559652227099	1.02223970843611	0.09552831007772
C	3.24329280518949	2.41521898682172	0.09842466292299
C	3.08082127418732	3.61426466051738	0.10716752478814
C	2.95482121527171	5.02648111340299	0.11250465465811
C	4.11038496922699	5.83017290739304	0.09702485104696
C	4.0197207739371	7.2118339917282	0.1033897977885
C	2.77190072846635	7.82918674860203	0.12561430919277
C	1.61396016755864	7.06046442789084	0.14081363375243
C	1.70834640535412	5.67696000870164	0.13392992150107
Cl	0.23563965187599	4.73603503777525	0.15128001889834
N	4.63769816804739	0.43118540240423	0.00404950122534

C	4.3726094717039	-0.9169453599096	0.00275339912178
C	5.41381092855641	-1.86774500967712	-0.05791974959468
C	6.33850673965484	-2.64804869396992	-0.09477936529946
C	7.37924590389325	-3.61043920077132	-0.14486702095081
C	7.06923966210922	-4.98378394120632	-0.15096692615859
C	8.06568665238438	-5.94454355086841	-0.19828160868369
C	9.40379262003307	-5.56206090864462	-0.24140733352066
C	9.74093142527233	-4.21360747915416	-0.23681688301532
C	8.73947329347851	-3.25520386481271	-0.18899218143267
Cl	9.19792763780244	-1.56663955872839	-0.18417208357733
C	2.99805159195059	-1.14369913151278	0.1088028260156
C	2.22622267163434	-2.45694556961457	0.19727141061285
O	1.30285525150971	-2.49279642176137	1.04198683883599
O	2.616197830572	-3.35543021494618	-0.57483665404862
H	0.66083013652953	0.72808406427189	-0.75231554057714
H	0.86204107016981	1.23998294023843	0.93781471523881
H	0.48511920037063	-0.45863626518666	0.56991871361345
H	5.07731236347886	5.34440198299619	0.08060792567135
H	4.92314754424495	7.80810871742131	0.0915596375827
H	2.6940582377495	8.90894997192168	0.13112292184923
H	0.64077416277963	7.53240573061444	0.15760474617728
H	6.02808074640827	-5.27732190481831	-0.11709736124567
H	7.79816496363808	-6.99363585044674	-0.20157704605426
H	10.1878169324559	-6.30773079291912	-0.27834943807378
H	10.7772830524268	-3.90519623199944	-0.26986691968898

3.7. Cartesian coordinates of the molecule 5g

	X	Y	Z
C	1.2792786745942	0.43023446287363	-0.27216174318848

N	2.7242566963192	0.26072136904266	-0.18561071583697
C	3.64760048835983	1.27272733213307	-0.0498631520396
C	3.30869240621725	2.63921858029173	0.02039465129676
C	3.03712364901344	3.81683555754274	0.07736966385965
C	2.72333516762754	5.20108006864979	0.14090621963529
C	3.74113393708986	6.15861162145302	0.2802509548939
C	3.42268183737657	7.50691752974506	0.33919206048321
C	2.09964147404373	7.9317437878782	0.26271547255558
C	1.10065986775284	6.97580343795272	0.12569059913073
Cl	-0.5721196306158	7.49495233930823	0.02820077750456
C	1.38567553407928	5.62237666249032	0.0632120213664
N	4.88089385361743	0.79381242575098	0.01522180421467
C	4.74848191077385	-0.57215435089976	-0.06298620004656
C	5.87458946902666	-1.42300668756544	-0.04158670583066
C	6.86216499419894	-2.12266767530697	-0.02107977845746
C	8.00702287207525	-2.96506386914138	-0.0012614776218
C	7.87110521353382	-4.36048966059687	-0.0921815701523
C	8.99408748337547	-5.17376059121031	-0.07113623486015
C	10.2696805582396	-4.62794556019138	0.04033718226125
C	10.3940233200578	-3.24719393912068	0.12960604532442
Cl	11.9950582878343	-2.54084549343388	0.2703266945868
C	9.29231519876461	-2.40852552295987	0.110924924585
C	3.4038232309031	-0.92288316192691	-0.19122244922882
C	2.74650996438831	-2.2871192024044	-0.36444845212907
O	3.17664270170174	-3.18851218251139	0.38390835488161
O	1.86067379993591	-2.34942270137927	-1.24529346428514
H	1.04344591747657	1.47987220459902	-0.11606430495614
H	0.78929947376862	-0.16501464912497	0.49679602352164
H	0.9244970866374	0.10899558405737	-1.24852900560196

H	4.7722195159731	5.83656044863907	0.34066789642259
H	4.21096232835528	8.241379200668	0.44621897959025
H	1.85190256655995	8.98346547448148	0.30877670229197
H	0.59264821850216	4.89552084701008	-0.04415535731155
H	6.88381502564676	-4.79467363543139	-0.17895440901811
H	8.88088345729937	-6.2483541062733	-0.14174736057224
H	11.1452843050443	-5.26245339107025	0.05703511460927
H	9.41359914445156	-1.33647155401881	0.18160023812145

3.8. Cartesian coordinates of the molecule 5h

	X	Y	Z
C	1.20062500404572	0.45526779541441	-0.07704511147867
N	2.632851269591	0.24389268044115	0.10090054788296
C	3.57706428273934	1.24073846658319	0.13401905653301
C	3.260407858396	2.61006838600702	0.03694314072189
C	3.00603815597872	3.79099913688721	-0.05476339626365
C	2.70588112649269	5.15611184064531	-0.1718611080638
C	3.57716644135721	6.22480018870774	-0.22071701560785
C	2.91855742013521	7.47084740753845	-0.34632115424118
C	1.55728883752912	7.35136301664324	-0.39230527356431
S	1.05576921418533	5.71347001251253	-0.28215205927886
N	4.80156551237339	0.74597028122054	0.2447984338681
C	4.64230407922348	-0.61951630737929	0.25841386124835
C	5.75438790603827	-1.47892213942332	0.38797945501924
C	6.73675431177657	-2.17911309299612	0.50298619108021
C	7.86837423232271	-3.00141487459721	0.62730990759026
C	7.93333225187684	-4.37897166266295	0.65415039831581
C	9.25403799335927	-4.87210097405641	0.78922359801499
C	10.1894203410421	-3.87840750687894	0.86454300935543

S	9.47002186649192	-2.32074527385919	0.77279071324341
C	3.29010197180803	-0.95500363223653	0.16817241596206
C	2.62076763743463	-2.32493620699697	0.1897902998288
O	1.54988916511541	-2.40618185294721	0.83443718181954
O	3.22882842957203	-3.22164411254765	-0.42898268764572
H	0.885339237299	1.30892843726076	0.52073379026895
H	0.6859115566764	-0.44075038723887	0.25505292722598
H	0.97677612010903	0.65055967784071	-1.12727083614899
H	4.65013118759622	6.10391727862822	-0.1683422614941
H	3.43391263443611	8.41982317099345	-0.40115303592652
H	0.82043717371495	8.13478599995374	-0.48582361398664
H	7.0541385146678	-5.00358870415728	0.57860138469439
H	9.50097320409103	-5.92424697337171	0.82852919977287
H	11.2596760625243	-3.97367907592806	0.968431041254

3.9. Cartesian coordinates of the molecule 5i

	X	Y	Z
C	1.27023782487672	0.46382975947091	0.41044939725525
N	2.70826557041247	0.25762680132169	0.28216248441358
C	3.63335156374368	1.2564788214868	0.10304156026509
C	3.29798870645872	2.62581213429754	0.07163333310236
C	3.02325154605919	3.80415354888932	0.04141471914225
C	2.69541764411138	5.18064854941663	0.01137009640247
C	1.35753982977215	5.69090259718371	0.12392072130043
C	1.30882807071373	7.04874976074077	0.06621713007835
S	2.8781393000256	7.73253693814748	-0.12473498917073
C	3.62184808185935	6.19182458638653	-0.12960574373814
N	4.85437877168236	0.76403078459896	-0.04748644827171
C	4.70764949013292	-0.60202353631773	0.01315108041087

C	5.82671657463886	-1.45880654138223	-0.08942567037331
C	6.81848557222063	-2.14742528371144	-0.17892316254649
C	7.9556067725781	-2.98639003802523	-0.28722638541584
C	7.89805995900475	-4.42168478627129	-0.2484855703295
C	9.12203549662538	-5.00176910724261	-0.37278284239557
S	10.3674061972708	-3.8239478079154	-0.53980156744567
C	9.24745648322661	-2.53155270699612	-0.44206795986368
C	3.36956254373494	-0.9398137085968	0.21923479265638
C	2.72466619245632	-2.30873986798523	0.40673153481634
O	3.17818940865384	-3.21481631562744	-0.32131515880808
O	1.82871873684465	-2.3808625809322	1.27958784300219
H	1.08504047203466	1.32293984441785	1.05284426713488
H	0.84173736071826	-0.42998501901845	0.85267313844933
H	0.82787546206157	0.6480685034018	-0.57020926731629
H	0.48816110634238	5.05940319722378	0.24084447573515
H	0.44069814758972	7.68721869242687	0.12484003494878
H	4.69133408553316	6.09467748545066	-0.23349815337639
H	6.97684850758465	-4.97545798755077	-0.13271193444985
H	9.36359428555878	-6.05363641380468	-0.37623454415487
H	9.58197023547347	-1.50756930348368	-0.50187021145762

3.10. Cartesian coordinates of the molecule 5j

	X	Y	Z
C	1.31216370177363	-0.09282679504633	-0.58828015068596
N	2.72380832297518	-0.27299318710519	-0.27572146792718
C	3.65281181517922	0.73158957203182	-0.17555813228115
C	3.34997689274146	2.10478349947772	-0.34129217442417
C	3.11520592732042	3.28115700762524	-0.48105601166735
C	2.82232672661971	4.70040744860304	-0.6250690373837

C	1.89008448415724	5.24275205302799	0.48610317050002
C	1.6108405262615	6.71677289637445	0.32565141933958
C	2.41763220154221	7.6702946839132	0.95233561577203
C	2.17829963664873	9.03122031440993	0.78546205693297
C	1.12444489361988	9.46251701853409	-0.01495578409793
C	0.31253815438775	8.52303628946114	-0.64448174687692
C	0.55525169318694	7.16332858400947	-0.47426563028156
N	4.85854860558279	0.24775241401368	0.08374938354217
C	4.70598719631349	-1.11913978596073	0.13424593441579
C	5.80966504592603	-1.96453741216502	0.41843513550854
C	6.77640796852514	-2.6437441489742	0.67078893949901
C	7.92872494887901	-3.4821560545733	0.98445441102573
C	8.70919985369498	-3.04663228933351	2.24908306515756
C	9.38818703996179	-1.70504966366423	2.12166851027963
C	8.84375062825856	-0.55965256731143	2.70712952809406
C	9.46488625607594	0.67959941929535	2.57099806999144
C	10.6459539692479	0.79357282455995	1.84513794468957
C	11.202069081944	-0.34106720058796	1.2589727245744
C	10.5781249866502	-1.5761825961846	1.397626006576
C	3.37609401976954	-1.46545914743227	-0.0862543845191
C	2.68935303361917	-2.82453443503295	-0.07039959229009
O	1.54880577950184	-2.85295412755003	0.44711326984026
O	3.34548133667559	-3.76758980748554	-0.56033010721242
H	1.11312363566008	0.97211851708861	-0.67872915150198
H	0.70284392137944	-0.52707984350793	0.19925544862997
H	1.0704272738927	-0.58284391032461	-1.53149140296986
H	2.36362767452175	4.87756004995001	-1.60274298037037
H	3.76339718629998	5.25855379533135	-0.61577507599007
H	2.35700791674427	5.05638100869929	1.45514105367899

H	0.9537389516188	4.68162539281439	0.45934146692395
H	3.23846193243745	7.34290612060678	1.58115278180131
H	2.81322780593157	9.75441552847018	1.28373106106885
H	0.93459914556484	10.5213745662514	-0.14365473750231
H	-0.51385383939193	8.8486424824725	-1.26538871834199
H	-0.08645064219194	6.4388750681123	-0.96389109427087
H	8.6119376350239	-3.50129521233518	0.12943713389773
H	7.58564743198213	-4.5114301913404	1.12809792950912
H	9.45863679067301	-3.81795475289276	2.44287702035144
H	8.02485087214888	-3.03626949724357	3.09952791831223
H	7.92642365947743	-0.63903514443872	3.27948625267384
H	9.02551131319714	1.55501852531949	3.03470940759996
H	11.1320700860187	1.75610708578144	1.74017229119186
H	12.1256039278609	-0.26450441503691	0.69702029452414
H	11.0229745941107	-2.45412898070768	0.94172713469276

3.11. Cartesian coordinates of the molecule 5k

	X	Y	Z
C	1.41266294021339	0.62769566351477	-0.33676474050193
N	2.82514794854575	0.26591810341715	-0.33312559698103
C	3.86408508458823	1.16101113487175	-0.35956819144082
C	3.67949430306334	2.5628847450306	-0.33354328597691
C	3.51996488105663	3.76154677322127	-0.30383731047897
C	3.33187101267758	5.18047806978153	-0.27478916260676
C	2.27830561701358	5.7885222712641	0.63609987600058
C	3.72359040938528	5.97944422935579	0.95807680690791
N	5.03278784202887	0.5389995189457	-0.42226300623695
C	4.73576769193658	-0.80391210937342	-0.46368161872569
C	5.76857358886098	-1.7750351332551	-0.50750163910166

C	6.68346525033322	-2.5660564069062	-0.52623348771574
C	7.76274403356963	-3.51089323129073	-0.56505446784037
C	8.10543519671632	-4.34479470093283	0.65820757853335
C	7.47855534480174	-5.00173760544208	-0.52986607381672
C	3.3567253192587	-0.99706738753623	-0.4079982138715
C	2.54572697939828	-2.28708464362874	-0.35183610970153
O	2.96157226217543	-3.21791500329493	-1.07167585878377
O	1.5602558190107	-2.28330534940594	0.42332409998389
H	1.25440913255434	1.47227746874023	0.33143082140578
H	0.84529458238945	-0.23054542374375	0.00899885437699
H	1.09720311024998	0.90581274371374	-1.34423205425408
H	3.47853987474339	5.68002833368639	-1.22564348562328
H	1.70725899344999	5.10532473335888	1.25029302908763
H	1.72169630625258	6.62610393443422	0.23689905281959
H	4.13582114401575	5.42634856727555	1.79132024445703
H	4.16887270242212	6.94959926425618	0.78110557163809
H	8.60665036845027	-3.20925036339247	-1.17506539469759
H	7.49369970195881	-4.2000635544064	1.53876915769923
H	9.15633206436209	-4.51849228145383	0.84991268485496
H	6.44228484461913	-5.3033379392394	-0.45460897682804
H	8.09622630049249	-5.62954932469056	-1.15869622561109

4. References

1. O'Boyle, N. M.; Banck, M.; James, C. A.; Morley, C.; Vandermeersch, T.; Hutchison, G. R. Open Babel: An open chemical toolbox. *J. Cheminf.* **2011**, *3*, 33. DOI:10.1186/1758-2946-3-33 ; The Open Babel Package, version 3.1.1 http://openbabel.org
2. Bannwarth, C.; Caldeweyher, E.; Ehlert, S.; Hansen, A.; Pracht, P.; Seibert, J.; Spicher, S.; Grimme, S. Extended tight-binding quantum chemistry methods. *WIREs Comput. Mol. Sci.*, **2020**, *11*, e01493.
3. Neese, F. The ORCA program system. *WIREs Comput Mol Sci* **2012**, 2, 73–78.
4. Becke, A. D. Density-functional exchange-energy approximation with correct asymptotic behavior. *Phys. Rev. A* **1988**, *38*, 3098–3100.
5. Lee, C.; Yang, W.; Parr, R. G. Development of the Colle-Salvetti correlation-energy formula into a functional of the electron density. *Phys. Rev. B* **1988**, *37*, 785–789.
6. Raghavachari, K. Perspective on "Density functional thermochemistry. III. The role of exact exchange". *Theor. Chem. Acc.* **2000**, *103*, 361–363.
7. Arnim, H.; Dmitrij, R. Development of new auxiliary basis functions of the Karlsruhe segmented contracted basis sets including diffuse basis functions (def2-SVPD, def2-TZVPPD, and def2-QVPPD) for RI-MP2 and RI-CC calculations. *Phys. Chem. Chem. Phys.* **2015**, *17*, 1010-1017.
